# Reference Correlation for the Viscosity of Helium

**DOI:** 10.1007/s10765-026-03816-5

**Published:** 2026-10-08

**Authors:** Marcia L. Huber, Daniel G. Friend

**Affiliations:** 1 Applied Chemicals and Materials Division, National Institute of Standards and Technology, 325 Broadway, Boulder, CO 80305 USA; 2 National Institute of Standards and Technology, 325 Broadway, Boulder, CO 80305 USA

**Keywords:** Correlation, Helium, Transport properties, Viscosity

## Abstract

We present a new wide-ranging correlation for the viscosity of normal helium (He I) based on critically evaluated experimental data as well as high-accuracy ab initio calculations. The correlation is designed to be used with densities from a high-accuracy Helmholtz equation of state, covering temperatures from 2.1768 K to 1500 K and pressures up to 2000 MPa. The estimated uncertainty at the 95 % confidence level depends on temperature and pressure, spanning from 0.002 % in the dilute gas limit (298.15 K, 0 MPa) to 10 % for the liquid phase at pressures from saturation up to 8 MPa. Finally, we provide comprehensive comparisons with experimental data and Python code to facilitate easy implementation.

**Supplementary Information:**

The online version contains supplementary material available at https://doi.org/10.1007/s10765-026-03816-5.

## Introduction

Helium is an important industrial fluid, with varied uses in industry such as a coolant in magnetic resonance imaging equipment, a buffer gas in helium–neon excimer lasers, an inert shielding gas in arc welding, a breathing gas for divers, and in semiconductor processing [1]. As a result of the passage of the CHIPS Act (Creating Helpful Incentives to Produce Semiconductors) of 2022 [2], there is increased interest in improving the calibration of the flow of gases used in semiconductor processing. The production of semiconductors uses many different gases, and flow meters are often calibrated with one gas, such as nitrogen, argon, or helium. Improved flow meter performance can be obtained by then determining calibration coefficients for other gases using gas-property data [3]. Viscosity is one of the properties used in the development of some flow meter models. It is the goal of this work to incorporate recent improvements in quantum-chemical ab initio computations that improve the representation of the dilute gas viscosity, and include new experimental data, to develop a wide-ranging correlation for the viscosity of helium that is valid in the liquid, gas, and supercritical phases.

Helium occurs as two different stable isotopes, ^3^He and ^4^He. In this work, we will address only ^4^He. We also restrict our study to normal helium (He I), and do not cover superfluid helium (He II) that forms in liquid ^4^He below about 2 K.

In 1969, Hanley and Childs [4] reviewed the literature and presented a very preliminary viscosity model valid for liquid, gas, and supercritical phases from 2 K to 50 K. In 1971, Tsederberg et al. [5] published comprehensive tables with correlating equations on the thermophysical properties of helium, including viscosity, but it is limited to temperatures above 0 °C. Vargaftik [6] published data tables compiled from various data sources for helium gas at 1 bar (0.1 MPa), at selected temperatures from 77 K to 1273 K at pressures up to 800 bar, and for the liquid from 2.186 K to 4 K, but no correlating equations are given. Stephan and Lucas [7] published a book on the viscosity of fluids, including helium. Their recommended tables for helium, however, are based on the compilation of Vargaftik [6]. In 1998, Donnelly and Barenghi [8] studied the properties of liquid helium, including both helium I and II that includes a very thorough bibliography of the viscosity of the liquid phase and tables of recommended values for the saturated liquid obtained by cubic spline fits of the evaluated data. Currently, a widely accepted comprehensive correlation for the viscosity surface of normal helium is that of Arp et al. [9] which was developed in 1998 and implemented in the NIST REFPROP database [10]. The 1998 publication is based on an earlier 1972 publication [11]. It is largely based on the work of Steward and Wallace [12] and the correlating equation has an uncertainty of 10 % for temperatures above 300 K; that work incorporates data only until 1972, providing an opportunity for improvement. Very recently, Yang et al. [13] presented a comprehensive review of models for the viscosity of helium over wide *p*–*T* ranges and identified the Arp et al. [9] model as the most reliable for dense and liquid phases.

Since 1972, there have been developments that can be used to improve the helium viscosity correlation presently in use. Advances have occurred in ab initio calculations that allow accurate calculation of the viscosity in the zero-density limit. For helium, the uncertainty in the calculated absolute viscosity of the dilute gas is almost two orders of magnitude smaller than uncertainties for existing experimental measurements [14]. Recently Xiao et al. [15–17], developed reference correlations for the dilute gas transport properties of ten fluids, including helium; these are based on the ab initio calculations. Additionally, new experimental data are available [18–40] that can be evaluated and possibly used to develop an improved correlation.

The first step in the development of a new correlation is the assembly and critical assessment of available literature data to determine the best data for use in correlation development. We identify two categories of experimental data: primary data, for use in the development of the correlation, and secondary data, used only for comparison purposes. According to the recommendation adopted by the Subcommittee on Transport Properties (now known as The International Association for Transport Properties) of the International Union of Pure and Applied Chemistry, the primary data are identified by a well-established set of criteria [41]. These criteria have been used successfully to establish standard reference values for the viscosity and thermal conductivity of fluids over wide ranges of conditions, with uncertainties in the range of 1 %. However, such a narrow definition of primary data greatly limits the range of data representation. Consequently, within the primary data set, for this work, it is also necessary to include results that extend over a wide range of conditions, albeit with higher uncertainty. Only data which are consistent with other lower uncertainty data or with theory are included in this expanded primary dataset. In all cases, the uncertainty claimed for the final recommended data must reflect the estimated uncertainty in the primary information.

## The Viscosity Correlation

The viscosity *η* can be expressed [42] as

1 $$ \eta \,\left( {\rho ,{\mathrm{T}}} \right)\, = \,\left( {\eta_{0} {\kern 1pt} \left( {\mathrm{T}} \right)\, + \,{\Delta }\eta_{{{\mathrm{res}}}} \left( {\rho ,{\mathrm{T}}} \right)} \right)\, \cdot \,{\Delta }\eta_{{\mathrm{c}}} {\kern 1pt} \left( {\rho ,{\mathrm{T}}} \right) $$

where *ρ* is the density and *T* is the absolute temperature. In the current study, *ρ* is in mol·L^−1^ (also expressed equivalently as mol·dm^−3^ in some usages), *T* is in K, and the viscosity is in μPa·s. Mass densities, kg·m^−3^, are used in some parts of our discussion, when that is the more common usage; using the He molar mass in Table 2, 1 mol·dm^−3^ is equivalent to 4.002602 kg·m^−3^. The first term, *η*_0_(*Τ*) = *η*(0,*Τ*), is the contribution to the viscosity in the dilute gas limit, where only two-body molecular interactions occur. The second term Δ*η*_res_(*ρ*,*T*) is often called the residual term and represents the contribution of all other effects to the viscosity of the fluid at elevated densities, including many-body collisions, molecular–velocity correlations, and collisional transfer. It can also be expressed as

2 $$ \Delta \eta_{{{\mathrm{res}}}} (\rho ,T) = \eta_{1} (T)\rho + \Delta \eta_{{{\mathrm{high}}}} (\rho ,T) $$

where *η*_1_(*Τ*) *ρ* is a linear-in-density term, known as the initial density dependence term, and all higher-order contributions are contained in the term Δ*η*_high_(*ρ,Τ*). The initial density dependence term often can be separately determined with the Rainwater–Friend theory [43–45] for the transport properties of moderately dense gases or can be found by fitting the full term Δ*η*_res_ to experimental data [46]. The last factor, Δ*η*_c_(*ρ,Τ*), is a dimensionless multiplicative term that accounts for the critical enhancement of the viscosity. This is due to long-range density fluctuations that occur in a fluid near its critical point, which contribute to divergence of the viscosity at the critical point. The critical enhancement term for viscosity is significant only in the region very near the critical point, as shown in Vesovic et al. [47] and Hendl et al. [48]. Experimental data for the apparent viscosity of helium are presented graphically by Agosta et al. [49] and the behavior is well described by the theoretical mode-coupling approach of Olchowy and Sengers [50]. To interpret the experimental measurements, gravitationally induced density gradients in the experimental cell were accounted for by Agosta et al. [49] and a near-critical background function for viscosity was developed. However, because the critical enhancement is important in only such a small range, and theoretical interpretation remains difficult, Δ*η*_c_(*ρ,Τ*) is often set to one, and the critical effect on viscosity is ignored. In this work, we will set the critical enhancement term to one and not discuss it further.

**Table 1:** Viscosity measurements of helium

First author	Publ year	Technique	Purity (%)	Uncertainty (%)	No. data	Temperature range (K)	Pressure range (MPa)	Density range (mol·L^−1^)
**Primary data**								
Kochan-Eilers [25]^a^	2026	VW	99.999	0.3	28	293	1.2–21	0.49–7.85
Sakoda [27]	2015	VW	99.99995^b^	1.4	20	296–573	0.10–0.72	0.11–0.29
Herrmann [21]^a^	2014	VW	99.999	0.25–0.3	126	293	0.12–25.3	0.05–9.27
Seibt^a^ [28]	2009	VW	99.996	0.25–0.3	96	293	0.12–30	0.05–10.84
May [26]	2007	Cap	99.999	0.16	2	298	0–0.1	Dilute gas
Evers [18]	2002	RotCyl	99.996^c^	0.15–0.4	44	293–348	0.12–9.5	0.05–3.74
Wang [33]	1990	TC	NA	4	14^d^	2.2–4.4	0.005–0.119	Sat. liquid
Strehlow [25, 29]^a,e^	1987/2026	OD	99.999^b^	0.2–0.4	16	299–688	0.08–0.18	Dilute gas
Vogel [25, 32]^a,e^	1984/2026	OD	99.999^b^	0.2–0.4	20	294–648	0.04–0.18	Dilute gas
Kestin [24]	1983	OD	NA	0.3	5	298–473	0.1	Dilute gas
Karnus[22]	1979	VW	NA	1.5–3	77^f^	14–60	0.07–11.4	0.18–44.6
Kestin [23]	1978	OD	99.995	0.1–0.3	9	298–778	0.1	Dilute gas
Gough [20]	1976	Cap	99.8^ g^	1–1.7	11	120–320	0.1	Dilute gas
Vermesse [31]	1976	Cap	NA	0.5^ h^	22	298	30–601	10.8–75.3
Goodwin [19]	1974	VW	NA	5–6	48^d^	2.3–4.20	0.45–2.5	33.68–43.78
Tsederberg [30]	1974	OD	NA	2	62	79.6–273	0.1–40	0.04–35.27
Kestin [56]	1972	OD	99.9995	0.1	8	298–973	0.1	Dilute gas
Webeler [57]	1972	TC	NA	NA	8^d,f^	2.18	0.005	36.48
Kestin [58]	1971	OD	NA	0.2	30	298	0.12–10	0.05–3.70
Steward [12]	1971	TC	NA	2.5–8	121	3.99–20.1	0.0049–10	0.005–50.91
Dawe [59]	1970	Cap	99.995^f^	1	15	293–1600	0.01–0.04	Dilute gas
Kalelkar [60]	1970	OD	NA	NA	9	298–1121	0.1	Dilute gas
Clarke [61]	1969	Cap	99.9^ g^	1	10	77.5–374	0.02–0.06	Dilute gas
Gracki [62]	1969	Cap	99.995	0.1–0.2	35	183–298	0.47–17	0.15–24.01
Guevara [63]	1969	Cap	NA	0.4	22	1100–2149	0.1	Dilute gas
DiPippo [64, 65]	1967,68	OD	NA	0.05^ h^	24	297–673	0.03–0.18	0.006–0.068
DiPippo [66]	1967	OD	NA	0.1	5	303	0.11–2.3	0.04–0.9
Kao [67]	1967	Cap	99.999^c^	0.14	53	183–323	1–51	0.1–5
Reynes [68]	1966	Cap	99.99	NA	36	373–473	7.1–83	1.78–20.78
Iwasaki [69]	1963	OD	99.99	0.2	9	293–303	0.1–5.3	0.16–8.38
Kestin [70]	1963	OD	99.989	0.5	34	296–521	0.14–12	0.03–3.02
Flynn [71]	1963	Cap	99.99	0.1	23	223–373	2.2–17.8	1.2–8.4
Makavetskas [72]	1963	Cap	NA	3.5	35	284–918	4–51	0.64–17.48
Kestin [73]	1959	OD	99.995	0.2–0.3	11	293	0.04–6.3	0.02–2.5
Coremans [74]	1958	OD	NA	2–3	28	20.4–77.8	0.01–0.05	Dilute gas
Ross [75]	1957	Cap	99.5^ g^	1	28	223–298	6.9–69	2.69–26.18
Becker [76]	1955	OC	NA	1	5	14.1–20.3	0.00033	Dilute gas
**Secondary data**								
Howald [77]	1992	TC	NA	NA	^f^	0.5–3	Saturation	Sat. liquid
Lukin [36]	1983	Cap	NA	0.3	23	76.5–293	0.1	Dilute gas
Tanaka [39]	1980	RB	99.99	1	17	298–323	0.1–1.1	0.018–0.428
Vidal [40]	1980	Cap	NA	0.5	7	298	0.1–600	0.04–75.3
Kestin [35]	1977	OD	99.995	0.1–0.2	9	298–678	0.1	Dilute gas
Kestin [78]	1974	OD	99.99	0.1–0.3	8	298–973	0.1	Dilute gas
Maitland [37]	1974	Cap	99.995	1–1.5	6	295–1533	0.1	Dilute gas
Rakshit [38]	1974	OD	NA	1	4	237–308	0.1	Dilute gas
Borisov [34]	1973	Cap	NA	0.2	1	300	0.1	Dilute gas
Kestin [79]	1972	OD	99.9995	0.1–0.3	6	298–973	0.1	Dilute gas
Kestin [80]	1968	OD	99.995	0.05^ h^	6	303	0.11–2.3	0.04–0.89
Richardson [81]	1968	Cap	NA	0.5	5	293	0.19–7.2	0.08–2.84
Rudenko [82]	1968	OC	NA	2	99	14.2–31	0.1–10	0.39–43.14
Kestin [83]	1966	OD	99.995	0.1	20	303	0.1–2.5	0.04–0.98
Golubev [84]	1965	Cap	99.914	0.5	103	273–523	0.1–78	0.02–4.11
Webeler [85]	1965	TC	NA	NA	4^d^	2.180–2.195	0.005	Sat. liquid
Kestin [86]	1964	OD	99.989	0.1	17	293–303	0.11–6.4	0.03–2.56
Stevenson [87]	1963	RB	99.9	0.1–0.4	3	278–363	0.1	Dilute gas
Thornton [88]	1962	Cap	NA	1	2	291	0.093	Dilute gas
Thornton [89]	1961	Cap	NA	1	1	291	0.093	Dilute gas
Thornton [90]	1960	Cap	NA	1	1	291	0.093	Dilute gas
Welber [91]	1960	TC	NA	NA	^i^	1.4–4	Saturation	Sat. liquid
Kestin [92]	1959	OD	99.989	0.05	11	293	0.042–6.3	0.02–2.49
Kestin [93]	1959	OD	99.9976	0.2–0.5	16	298–512	0.1–14	0.02–5.0
Luker [94]	1959	Cap	99.95	1.5	30	328–682	0.82–13	0.18–3.15
Rietveld [95]	1959	OD	NA	1–3	5	20.4–293	0.0053–0.0077	Dilute gas
Zinovieva [96]	1958	Cap	NA	NA	14^d^	2.2–4.2	0.005–0.099	Sat. liquid
Eisele [97]	1958	OC	NA	20–30	17^d,f^	2.2–4.25	0.005–0.1	Sat. liquid
Kestin [98]	1958	OD	98	0.2	8	298	0.1–5.1	0.04–2.00
Taylor [99]	1957	OD	NA	NA	25^d^	2.185–3.95	0.005–0.078	Sat. liquid
Robinson [100]	1955	FWt	NA	10	25^f^	16–90	0.02–0.18	22.6–85.5
Becker [101]	1954	OC	NA	1	9^d^	2.22–4.20	0.005–0.099	Dilute gas
Kestin [102]	1954	OD	98	0.2	10	293	0.11–5.2	0.05–2.07
Rietveld [103]	1953	OD	NA	1	13	72–291	0.1	Dilute gas
Van Itterbeek [104]	1953	OD	NA	NA	45^d^	2.29–4.3	0–0.095	Dilute gas
Tjerkstra [105]	1952	Cap	NA	NA	22^d^	2.19–4.07	0.96–4.90	37.7–47.5
De Troyer [106]	1951	OD	NA	NA	14^d^	2.27–4.03	0.006–0.08	Sat. liquid
Bowers [107]	1950	Cap	NA	NA	33^d^	2.22–4.93	0.005–0.19	Sat. liquid
Johnston [108]	1942	OD	NA	0.3–0.5	28	79.2–296	0.1	Dilute gas
Wobser [109]	1941	FBall	NA	NA	5	293–371	0.1	Dilute gas
Van Itterbeek [110]	1940	OD	99	NA	4	14.6–290	0–0.1	Dilute gas
Van Itterbeek [111]	1940	OD	NA	NA	31	14.6–290	0–0.1	Dilute gas
Van Itterbeek [112]	1938	OD	NA	1	40^d^	4.23–90.3	0–0.1	Dilute gas
Keesom [113]	1938	OD	NA	NA	15^d^	2.18–4.02	0.005–0.083	Sat. liquid
Trautz [114]	1935	Cap	99.5	NA	3	90–273	0.1	Dilute gas
Wilhelm [115]	1935	OC	NA	NA	4	2.4–4.21	0.008–0.1	Sat. liquid
Trautz [116]	1931	Cap	NA	NA	4	293–550	0.1	Dilute gas
Trautz [117]	1934	Cap	NA	NA	10	293–523	0.1	Dilute gas
Trautz [118]	1930	Cap	99	NA	14	293–523	0.1	Dilute gas
Trautz [119]	1930	Cap	99	NA	14	292–1091	0.1	Dilute gas
Nasini [120]	1928	Cap	NA	NA	4	284–373	0.1	Dilute gas
Günther [121]	1924	SWP	NA	NA	16	15.5–273	0.016–0.1	Dilute gas
Ishida [122]	1923	OIL	NA	0.3	1	296	0.1	Dilute gas
States [123]	1923	CD	97–98	0.1	1	296	0.01	Dilute gas
Gille [124]	1915	Cap	NA	NA	6	273–373	0.007–0.10	Dilute gas
Vogel [125]	1914	OD	NA	NA	5	21–273	0.012–0.043	Dilute gas
Kamerlingh Onnes [126]	1913	Cap	NA	NA	16	15–294.5	0.05–0.06	Dilute gas
Rankine [127]	1910	Cap	99.875	NA	1	283	0.1	Dilute gas
Schmitt [128]	1909	Cap	NA	NA	7	80–458	0.075	Dilute gas

*NA* not available, *Cap* capillary, *CD* constant deflection, *Fball* falling ball, *FWt* falling weight, *OIL* oil drop, *OC* oscillating cylinder, *OD* oscillating disk, *OS* oscillating sphere, *RB* rolling ball, *RotCyl* rotating cylinder, *SWP* swinging plate, *TC* torsional crystal, *VW* vibrating wire, *VibPl* vibrating plates

^a^Experimental density used

^b^Volume percent

^c^Molar basis

^d^Data below 2.178 K not included

^e^Re-evaluated in 2025

^f^Presented graphically only

^g^Mass basis (if not stated no superscript given)

^h^Precision

^i^Viscosity not reported directly

In addition to performing literature searches and utilizing content of previous work, we made extensive use of the NIST ThermoData Engine [51] to identify data sources. Table 1 summarizes, to the best of our knowledge, all the available experimental measurements of the viscosity of helium reported in the literature. Table 1 also indicates the experimental method, the sample purity, uncertainty as reported by the original authors (unless noted otherwise), the number of measurements, as well as the range of temperatures, pressures, and densities. The abbreviations used for experimental methods are explained in a footnote below the table, and information about these methods can be found in [52]. The uncertainties tabulated in Table 1 are at the 95 % (*k* = 2) level; however, many authors failed to report *k* and, in such cases, we assumed *k* = 2. The uncertainties reported by the authors may be overly optimistic in some cases.

The correlation in Eq. 1 is formulated in terms of temperature and density. Experimental measurements of viscosity are generally performed at a known temperature *T* and pressure *p*, and it is necessary to obtain the density associated with the *T*, *p* state point. We use the Helmholtz equation of state (EOS) formulated by Ortiz-Vega et al. [53] to obtain density, after first converting all experimental viscosity temperatures to ITS-90 [54, 55] if necessary. The range of validity for this EOS is for the normal fluid (Helium I) up to 1500 K and 2000 MPa. Although this EOS is considered somewhat preliminary due to some known problems [53] such as incorrect ITS-90 temperature scale conversions that contributed to the inability of the EOS to satisfy the relationship between temperature and vapor pressure that forms part of the definition of ITS-90, it is the best one currently available. We also use the critical point associated with this EOS; the critical point and other constants for this EOS are given in Table 2. The uncertainties in calculated density below 50 K are 0.25 % at pressures up to 10 MPa, from 50 K to 200 K the uncertainties decrease linearly from 0.2 % at 50 K to 0.05 % at 200 K for all pressures up to 50 MPa. Between 200 K and 500 K, the density uncertainty is 0.03 % up to pressures of 40 MPa and 0.1 % between 40 MPa and 100 MPa. For all other states not listed here, the density uncertainties increase to 0.5 % in density. Additional details on the EOS are provided in reference [53].

**Table 2:** Critical point and fixed constants for the EOS of Ortiz-Vega et al. [53]

Property	Symbol	Units	Value
Critical temperature	*T*_c_	K	5.1953
Critical pressure	*p*_c_	MPa	0.22832
Critical density	*ρ*_c_	mol·dm^−3^	17.3837
Lambda-point temperature (lower)	*T*_lambda_	K	2.1768
Molar mass	*M*	g·mol^−1^	4.002602

**Fig. 1:**
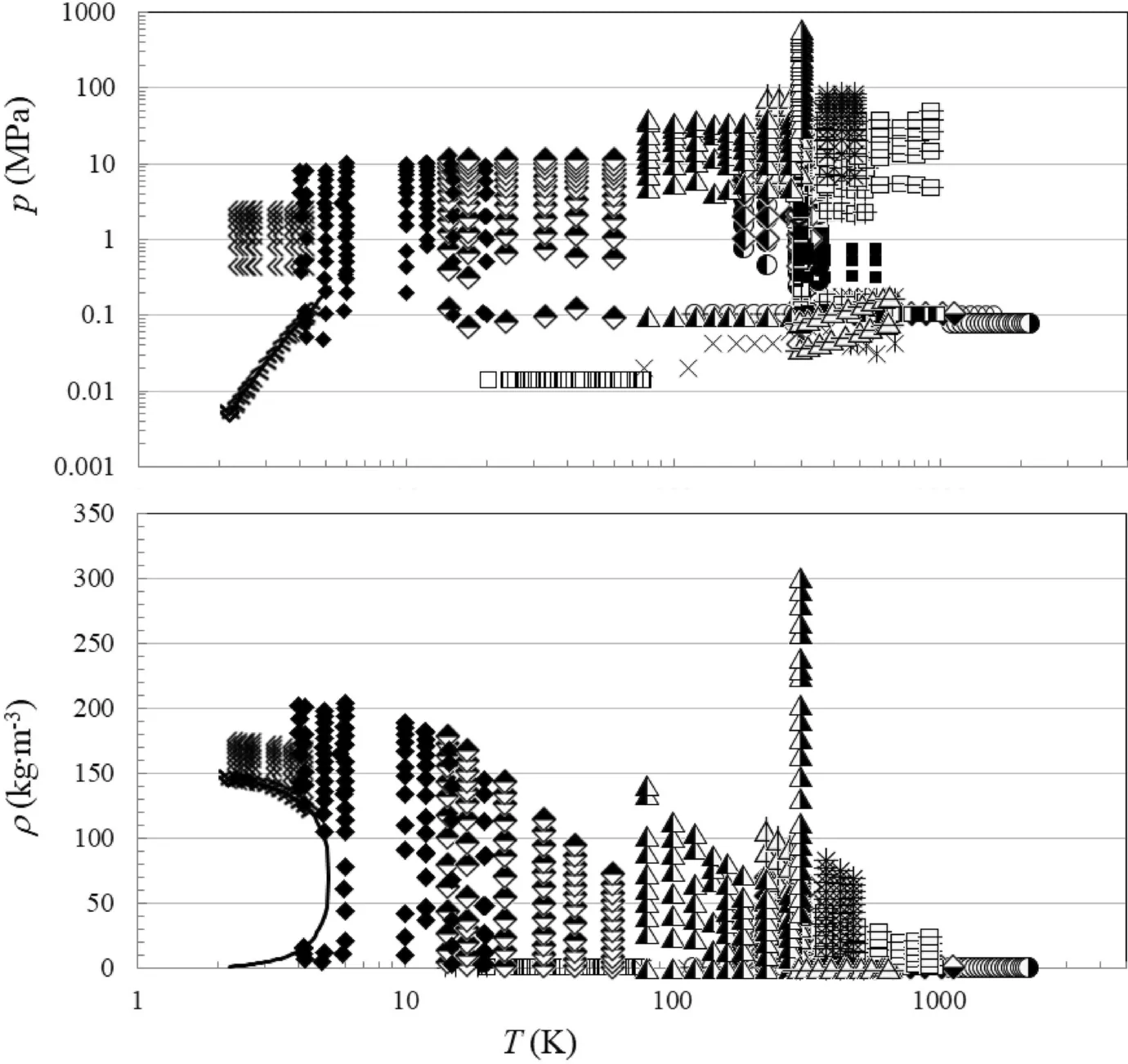
Temperature–pressure and temperature–density ranges of the primary experimental viscosity data for helium. (─) saturation curve, Becker and Misenta [76] (+), Clarke and Smith [61] (×), Coremans et al. [74] (□) Dawe and Smith [59] (○), DiPippo et al. [66] (), DiPippo and Kestin [64, 65] (), Evers et al. [18] (), Goodwin [19] (), Gough et al. [20] (), Gracki et al. [62] (), Guevara et al. [63] (), Herrmann [21] (), Iwasaki and Kestin [69] (), Kalelkar and Kestin [60] (), Kao and Kobayashi [67] (), Karnus et al. [22] (), Kestin and Leidenfrost [73] (), Kestin and Whitelaw [70] (), Kestin et al. [58] (), Kestin et al. [56] (), Kestin et al. [23] (), Kestin and Wakeham [24] (), Kochan-Eilers et al. [25] (), Makavetskas and Popov [72] (), May et al. [26] (), Reynes and Thodos [68] (), Ross and Brown [75] (), Sakoda et al. [27] (), Seibt et al. [28] (−), Steward and Wallace [12] (), Strehlow [29] (), Tsederberg et al. [30] (), Vermesse and Vidal [31] (), Vogel [32] (), Wang et al. [33] (), and Webeler and Allen [57] ()

In assembling a primary dataset, we first note that the dilute gas contribution to the viscosity will be based upon the ab initio calculations of Cencek et al. [14] and will not be based on fitting experimental data. However, we have included several datasets with the primary data at atmospheric pressure [20, 23, 24, 32, 56, 60, 63] over a wide range of temperatures primarily for comparison purposes. For groups that measured multiple datasets, we generally retained the newer sets or ones with a wide range of conditions for primary data. (For example, we retained Kestin et al. [58] as primary and moved Kestin and Leidenfrost [92, 93] to secondary data.) Where multiple datasets were available that covered the same *T*-*ρ* region, the data with the lowest uncertainties were selected for inclusion in the primary set. Although the vibrating wire measurements of Sakoda et al. [27] have a somewhat large uncertainty (1.4 %), we include them in the primary data as well, since they cover a wide temperature range. The measurements of Wang et al. [33] are included as primary to cover the saturated liquid phase. In addition, for the liquid phase, we follow the recommendations of Donnelly and Barenghi [8]. Their recommendations cover both above and below the lambda line, for points at saturation. Our correlation will only cover the region above the lambda line, so we use their recommended sources for temperatures above 2.1768 K, namely Goodwin [19], Webeler and Allen [57] and Wang et al. [33]. All other liquid sources were considered secondary, except for liquid points measured by Steward and Wallace [12]. The points of Steward and Wallace [12] are not at saturation and were not included in the review by Donnelly and Barenghi [8]. In addition, in the low-temperature region (below 60 K), we included both Steward and Wallace [12] and Karnus et al. [22] in the primary set, but classified Rudenko and Karnus [82] and Robinson [100] as secondary sources as they differ significantly from Steward and Wallace [12] and Karnus et al. [22] in their regions of overlap. Karnus et al. [22] suggested that there may be calibration errors in the earlier work [82]. Figure 1 illustrates the distribution of the primary data in the *T- p* and *T*- *ρ* planes. The saturation boundary as calculated by the EOS [53] is also shown.

### The Viscosity in the Dilute Gas Limit

**Fig. 2:**
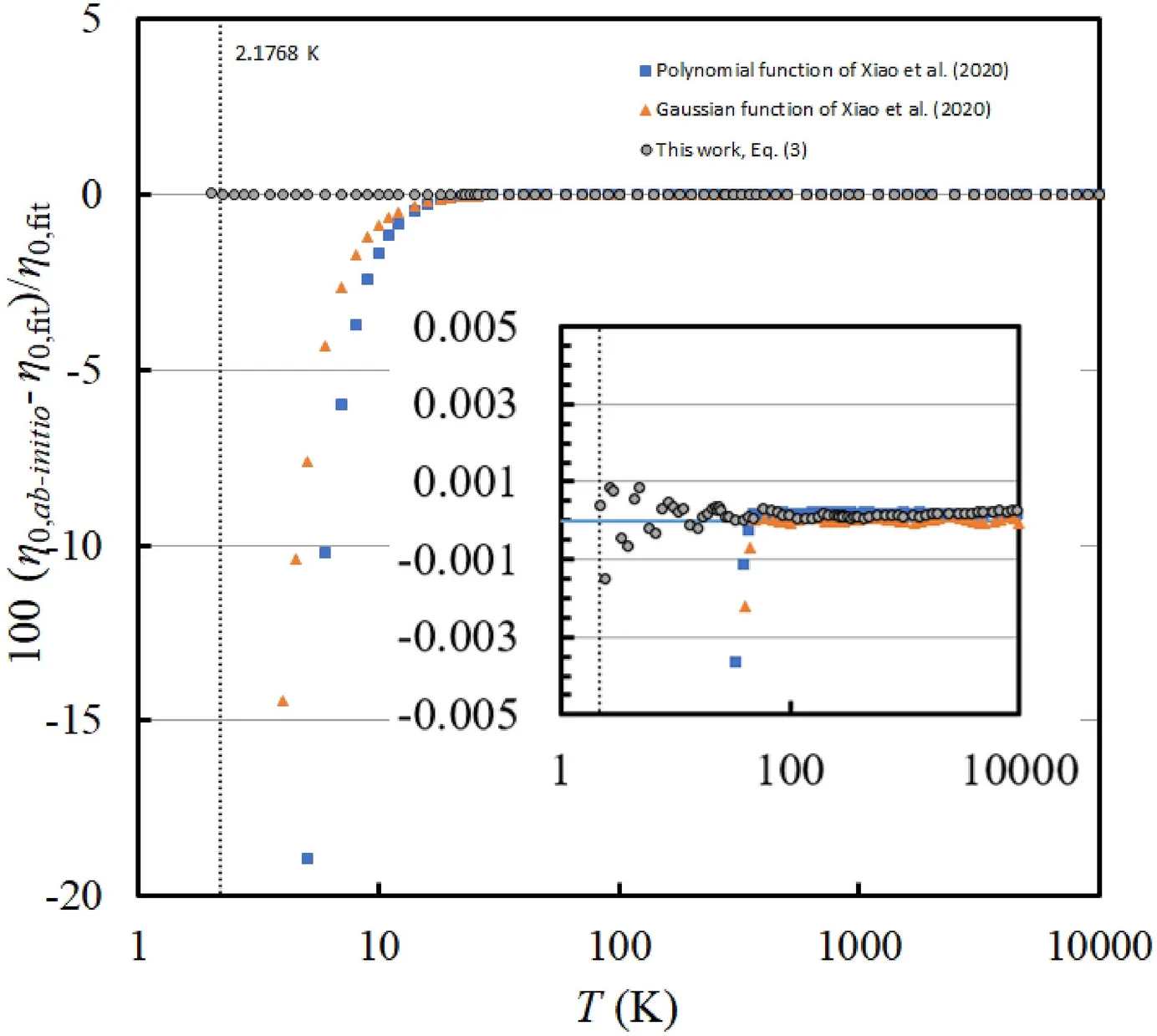
Percentage deviations of the correlations for dilute gas viscosity of Xiao et al. [15–17], the present work, and the ab initio values of Cencek et al. [14]

Theoretical calculations for the viscosity of dilute gases have been available since the solution of the Boltzmann equation by Chapman [129] and Enskog [130] more than a century ago. The kinetic theory approach requires knowledge of the intermolecular potential between two molecules of the fluid of interest. Considerable progress has been made in the kinetic theory approach and in estimates of the intermolecular potential: Garberoglio et al. [131] recently discussed how advances in ab initio calculations can provide thermophysical properties useful for metrology. These properties are calculated from first principles with rigorously propagated uncertainties. The ab initio calculations of Cencek et al. [14] provide an incredibly accurate value for the viscosity of helium at 25 °C and zero density, 19.8253 ± 0.0002 µPa·s (*k* = 1), that is much more accurate than can be determined experimentally. For example, the most accurate measurements of helium (see Ref. [26, 56]) have uncertainties on the order of 0.1 % to 0.2 %. In their Supplementary Material, Cencek et al. [14] provide values of the viscosity of helium in the limit of zero density at discrete temperatures and the associated uncertainty over the temperature range from 1 K to 10 000 K with an uncertainty ranging from 0.000 17 µPa·s at 1 K to 0.0016 µPa·s at 10 000 K. A correlation is needed to represent these values as a function of temperature. Xiao et al. [15–17] developed two different correlations for the dilute gas limit viscosity, *η*_0._ The first one presented in the manuscript is a polynomial form, and the authors state that a possible limitation to this functional form is that it does not extrapolate to very low-temperatures in a manner as expected by kinetic theory; they provide an alternative formulation in the supplemental information that can be used if one is concerned about the theoretical behavior in extrapolations to 0 K. The equation presented in the supplemental information is a Gaussian form and has more realistic behavior at very low temperatures. However, the representation of the ab initio values of Cencek et al. [14] declines for both equations at temperatures less than ~ 50 K, as shown in Fig. 2. At temperatures above 50 K, both correlations are in excellent agreement with the ab initio calculations and are well within the uncertainty of the ab initio values. However, for helium, our intent is to represent the complete fluid surface down to the lambda point, or 2.1768 K, for the dilute gas. Therefore, it was necessary to develop a new correlation that can be used over the complete range of temperatures from 2.1768 K to 10,000 K and represent the ab initio values to within their uncertainty. The new correlation for the dilute gas limit of the viscosity of helium is valid from 2 K to 10,000 K and is expressed as a rational polynomial

3 $$ \eta_{0} (T) = \eta_{0} (298.15{\kern 1pt} \;{\mathrm{K}})\left[ {\frac{{\sum\limits_{i = 0}^{11} {a_{i} (T^{{\mathrm{S}}} )^{i} } }}{{\sum\limits_{i = 0}^{9} {b_{i} (T^{{\mathrm{S}}} )^{i} } }}} \right], $$

where *T*^S^ is a scaled temperature *T*^S^ = *T*(K)/100, *η*_0_ (298.15 K) is the reference value at 298.15 K from Cencek et al. [14], 19.8253 ± 0.0002 µPa·s, and the coefficients are shown in Table 3. As shown in Fig. 2, the agreement with Cencek et al. [14] is very good; it is within the tabulated uncertainties of Cencek et al. [14] for the range of temperatures from 2 K to 10,000 K except for a single point at 2 K.

**Table 3:** Coefficients for the dilute gas viscosity Eq. 3

*i*	*a*_*i*_	*b*_*i*_
0	1.30095 × 10^–3^	3.48985 × 10^–2^
1	− 1.01318 × 10^–1^	1.40214 × 10^0^
2	6.71201 × 10^0^	2.03716 × 10^1^
3	− 6.22055 × 10^1^	3.76085 × 10^2^
4	1.73434 × 10^3^	2.26351 × 10^4^
5	1.90407 × 10^4^	6.15385 × 10^4^
6	2.79896 × 10^4^	3.39238 × 10^4^
7	9.76572 × 10^3^	4.63112 × 10^3^
8	9.07758 × 10^2^	1.55338 × 10^2^
9	2.17672 × 10^1^	1.00000 × 10^0^
10	1.06293 × 10^–1^	–
11	2.05960 × 10^–5^	–

### The Initial Density Dependence for Viscosity

The theory for the initial density dependence of transport properties (i.e., the slope of a viscosity versus density plot at a fixed temperature in the dilute gas region) is less advanced than for the dilute gas itself. Kinetic theory shows that while the zero-density limit of the viscosity depends only on pair interactions, the linear-in-density term *η*_1_(*Τ*) *ρ* in Eq. 2 is dependent upon interactions among two and three molecules. A theory for *η*_1_ was developed by Rainwater and Friend [43, 44] by analyzing all types of collisions that can take place among two or three molecules; these collisions (or scattering events) serve to transfer momentum, contributing to viscosity according to fundamental kinetic theory. Three types of collisions were considered for realistic interparticle potentials, described very briefly as follows. Firstly, there is a non-locality expansion associated with two molecules that serve as the density expansion of the well-known dilute gas limit theory: in performing this expansion, care must be taken to ensure that collisions are not influenced by a third particle in the scattering trajectories. Only the “free” part of the two-particle phase space is considered in all integrations necessary to calculate the contribution. Secondly, three monomeric molecules can collide, within the scattering process; this phenomenon was calculated by treating the third molecule as “shielding” the two-particle collision. Finally, realistic potentials include an attractive well, and two molecules can be (metastably) bound for times greater than a mean collision time in the system. Thus, monomer–dimer collisions contribute to the linear-in-density term for viscosity; this third contribution is calculated by considering the system to be a low-density mixture of monomers and dimers, using dilute gas mixture theory.

The theoretical approach is described in some detail by Rainwater and Friend [43, 44] and their cited references. It is important to note that the calculations were only implemented by Rainwater and Friend for the simple Lennard–Jones 12–6 potential. (Calculations for repulsive potentials had been completed by others in earlier publications.) Further, the monomer–dimer potential was also considered to be a spherically symmetric Lennard–Jones potential, with adjusted values for the well depth (*ε*/*k*_B_) and length parameter (*σ*). Additionally, quantum contributions to the relevant scattering processes were not considered. In practice, the results of the theoretical approach have been used as a two-parameter corresponding states model, with *ε* and *σ* taken from the literature for dilute gases and the adjustment for the monomer–dimer potential regressed from experimental moderately dense gas viscosity data for a variety of fluids. Thus, to use this approach, a “universal” expression for *η*_1_(*Τ*) is postulated, with only values for *ε* and *σ* needed to describe the fluid of interest.

Subsequently, Najafi et al. [132] introduced more realistic potential functions for noble gases and completed the calculations of Rainwater and Friend [43, 44] with these potentials. They presented a new generalized fit for the reduced second viscosity virial coefficient of noble gases. In addition, they presented a specific correlation for helium, based on the HFD-B3-FcI1 potential function of Aziz, Janzen, and Moldover [133]. Bich and Vogel [45, 134] used the Rainwater–Friend theory, but considered a more expansive set of moderately dense gas viscosity data to adjust the Lennard–Jones parameters for the monomer–dimer term. They presented a correlation for the initial density dependence [45] that is valid over the range 0.5 < T* < 100 where T* is a scaled, or reduced temperature T* = *k*_B_*T*/ε with the Lennard–Jones energy scaling parameter *ε*/*k*_B_ for the pure fluid. Vogel et al. [135] extended this relationship to lower temperatures, 0.3 < T^*^ < 100, and this empirical correlation has been used successfully for correlations for the viscosity surface of a variety of different fluids including refrigerants [136], aromatics [137], ammonia [138], and hydrocarbons such as butane [139].

**Fig. 3:**
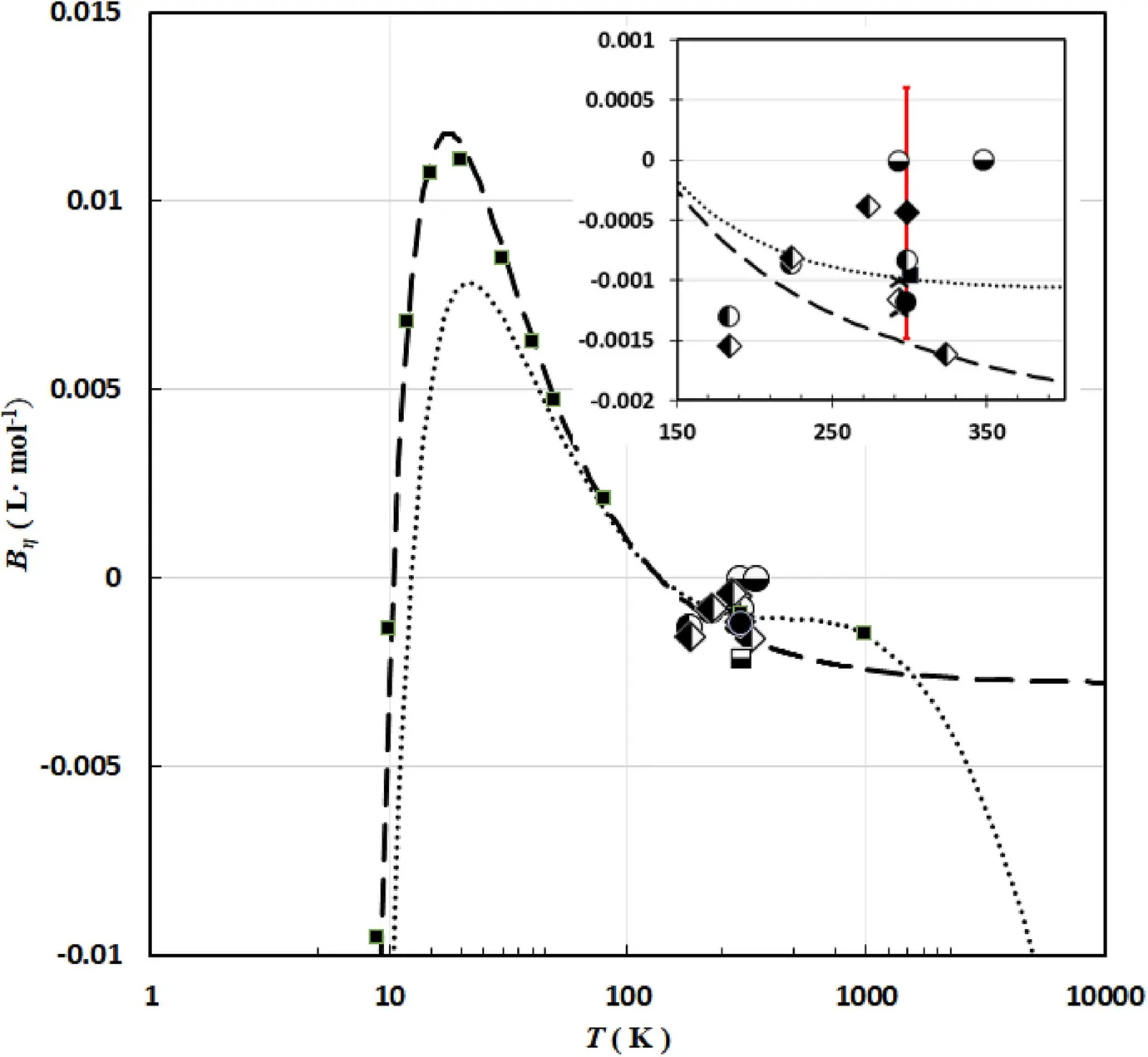
The second viscosity virial coefficient *B*_*η*_. Dotted line, Vogel et al. [135] correlation, dashed line, Najafi et al. [132] correlation, Berg and Moldover [141] (), Evers et al. [18] (), Gracki et al. [62] (), Herrmann [21] (), Kao and Kobayashi [67] (), Kestin and Leidenfrost [73] (), Kestin et al. [58] (), Kochan-Eilers et al. [25] (), Rainwater–Friend theory (),Vogel [28, 142] (). The vertical line at 298.15 K shows the uncertainty of the recommended value of Berg and Moldover [141] at *k* = 2

For the present study of helium, we first attempted to fit the primary data to Eq. 1 without specifically calculating a separate contribution for the initial density dependence term, as we recently did for the viscosity of nitrogen [140]. This was largely unsuccessful, since there are very few low-uncertainty experimental data points at low temperatures and low densities that could be used in fitting. Hence, we decided to incorporate a correlation with known behavior for the linear-in-density contribution to guide the behavior of the full correlation at low density. As shown in Fig. 3, for moderate temperatures, the Najafi et al. [132] correlation and the Vogel et al. [135] correlation for the second viscosity virial coefficient *B*_*η*_, defined by Eq. 5 below, are very similar. (These also agree well with the original calculations of Rainwater and Friend which were tabulated at discrete points in Ref. [44]). The correlation of Vogel et al. [135] is only intended for use up to T* = 100. Using helium LJ parameters *σ* = 0.263 nm, and *ε*/*k*_B_ = 10.00 K as given in Bich and Vogel [134], the upper limit on the Vogel correlation is 1000 K, and the behavior starts to deviate significantly from the correlation of Najafi et al. [132] above that limit. In the region where there are experimental data, the two correlations are similar. The values for Najafi et al. [132] in Fig. 3 are computed using their helium parameters (not the generalized correlation) and with scaling parameters [132] *σ* = 0.2641 nm, and *ε*/*k*_B_ = 10.956 K. As shown in Fig. 3, the two models differ at low temperatures and at very high temperatures. For regions of practical importance, the two correlations are very similar, and both agree with available experimental data [18, 21, 25, 28, 58, 62, 67, 73] and the recommended value of Berg and Moldover [141] as shown in Fig. 3. Values for experimental data were either taken directly from the literature reference [58, 62, 73] or obtained from the experimental data [18, 21, 25, 28, 67] by first (if necessary) correcting to the nominal temperature using the temperature dependence from our correlation of Cencek et al. [14] as given in Eq. 3 and then determining the value of *B*_*η*_ from a fit of the viscosity as a function of density along the nominal isotherm.

Vogel [142] also provided a recommended value for the viscosity of helium in the limit of zero density and the initial density dependence at 298.15 K, as needed for calibration of oscillating disk viscometers. He recommended the zero-density value of Cencek et al. [14] and an initial density dependence (∂*η*/∂*ρ*)_*T*_ [≡ *η*_1_(*T*) in Eq. 4] of − 5.878 nPa·s·m^3^·kg^−1^, or equivalently a value of *B*_*η*_ of − 2.965 × 10^−4^ m^3^·kg^−1^ (or − 0.001187 L·mol^−1^ in the units of Fig. 3). Vogel [142] obtained the initial density dependence from evaluating the data of Seibt et al. [28], combining the data from three different series of measurements after first correcting to 298.15 K. The Vogel [142] value is quite a bit more negative than the recommended value of Berg and Moldover [141] (− 1.1 × 10^−4^ m^3^·kg^−1^ ± 1.3 × 10^−4^ m^3^·kg^−1^ or 0.00044 L·mol^−1^), but lies within the *k* = 2 uncertainty of Berg and Moldover [141] as shown on the inset. The Vogel [142] value also is in closer agreement with the most recent data of Kochan-Eilers et al. [25] and Herrmann [21].

While the contributions by Vogel et al. [135, 142] and by others are compelling, we selected the correlation of Najafi et al. [132] in the current study for the first density correction (rather than the one from Vogel et al. [135]) primarily because of its better extrapolation behavior at very high temperatures, since the EOS is valid up to 1500 K. Thus for the initial density dependence, we use [132]

4 $$ \eta_{1} (T) = B{}_{\eta }(T)\eta_{0} (T), $$

5 $$ B_{\eta } (T) = N_{{\mathrm{A}}} \sigma^{3} B_{\eta }^{*} (T^{*} ), $$

6 $$ B_{\eta }^{*} (T^{*} ) = \sum\limits_{i = 0}^{6} {c_{i} (T^{*} )^{ - i} } , $$

where the coefficients *c*_i_ from Najafi et al. [132] are given in Table 4, *N*_A_ is the Avogadro number, T* is the scaled temperature T^*^ = *k*_B_*T*/*ε*, and* η*_0_ is the dilute gas viscosity given by Eq. 3.

**Table 4:** Parameters for the reduced second viscosity virial coefficient $B_{\eta }^{*}$, Eq. 6 [132]

*ε*/*k*_B_ = 10.956 K, *σ* = 0.2641 nm	
*i*	*c*_*i*_
0	− 0.2526
1	3.109
2	0.9481
3	− 5.5147
4	2.766
5	− 0.7225
6	− 0.07534

### The Viscosity Residual Term

The residual viscosity term Δ*η*(*ρ*,*T*) represents the contribution of all other effects to the viscosity of the fluid at elevated densities including many-body collisions, molecular–velocity correlations, and collisional transfer. Because there is little theoretical guidance concerning this term, its evaluation is based entirely on experimentally obtained data. A modification of an in-house software package developed for multi-property nonlinear fitting of equations of state allows consideration of multiple functional forms, numbers of terms, weighting of data, constraints of types of exponents, etc., and was used previously in our work on the viscosity of nitrogen [140]. In this work, we used the totally empirical fixed form

7 $$ \Delta \eta_{{{\mathrm{high}}}} (T_{{\mathrm{r}}} ,\rho_{{\mathrm{r}}} ) = \sum\limits_{i = 1}^{13} {N_{i} T_{{\mathrm{r}}}^{{t_{i} }} } \rho_{{\mathrm{r}}}^{{d_{i} }} $$

where *T*_r_ is the reduced temperature *T*/*T*_c_, *ρ*_r_ is the reduced density *ρ*/*ρ*_c,_ and *N*_*i*_, *t*_*i*_, and *d*_*i*_ are parameters (not restricted to integers or simple fractions) determined by fitting the primary experimental data (Table 1) with Δ*η*_high_ expressed in μPa·s. Within the fitting process for Δ*η*_high_, the dilute gas term (Eq. 3) and the linear-in-density term (Eqs. 4–6) were first subtracted from the primary experimental data. Non-integer exponents on density were allowed above 1.25, and positive temperature exponents were not permitted. During the fitting process, constraints were implemented to ensure that the vapor phase viscosity near saturation did not become negative. Weights were adjusted manually to represent the datasets to near or within their estimated uncertainties. Initial fits were done with 10 terms since that was sufficient for nitrogen [140], but we found it necessary to increase the number of terms to 13 for helium in order to fit the primary data well and obtain good extrapolation behavior. The resulting coefficients are given in Table 5.

**Table 5:** Coefficients *N*_*i*_, *t*_*i*_ , and *d*_*i*_ of Eq. 7

*i*	*N*_*i*_	*t*_*i*_	*d*_*i*_
1	123.2	− 0.418	1.303
2	62	− 0.24	1.66
3	36.82	− 5.352	1.253
4	− 39.3	− 5.52841	1.303
5	− 141	− 0.744	1.5
6	− 105	− 0.081	1.25
7	79	− 1.34	1.647
8	− 87.5	− 0.07	1.34
9	105.4	0	1.25
10	7.03	− 5.898	1.413
11	27.9	− 0.395	3.02
12	− 36	− 0.38	2.8795
13	− 25.7	− 1.9	1.72

The final expression for the viscosity of helium is then

8 $$ \eta (\rho ,T) = \eta_{0} (T) + \eta_{1} (T)\rho + \Delta \eta_{{{\mathrm{high}}}} (\rho_{{\mathrm{r}}} ,T_{{\mathrm{r}}} ) $$

where *η*, *η*_0_ , and Δ*η*_high_ are in μPa·s, *ρ* is in mol·L^−1^, and *η*_1_ is in μPa·s·L·mol^−1^ with Eq. 3 for *η*_0_, Eqs. 4–6 for *η*_1_, and Eq. 7 for Δ*η*_high_.

### Comparison with Data

Table 6 presents comparisons of the primary data with the present correlation, Eq. 8, while Table 7 gives comparisons of the secondary data. Comparisons with the correlation of Arp et al. [9] are also given. We use the following expressions for the percent deviation (PCT), average absolute relative deviation (AARD) and BIAS:

9 $$ {\mathrm{PCT}}_{i} = \frac{{100\;\Delta \eta_{i} }}{{\eta_{i} }} = \frac{{100(\eta_{\exp ,i} - \eta_{{{\mathrm{fit}},i}} )}}{{\eta_{{{\mathrm{fit}},i}} }}, $$

10 $$ {\text{AARD = }}\left( {\sum\limits_{i = 1}^{n} {\left| {{\mathrm{PCT}}_{i} } \right|} } \right)/n $$

11 $$ {\text{BIAS = }}\left( {\sum\limits_{i = 1}^{n} {{\mathrm{PCT}}_{i} } } \right)/n $$

where *n* is the number of data points, *η*_exp_ is the experimental value of the viscosity, and *η*_fit_ is the value calculated from the fitted correlation. The maximum deviation (positive or negative) is also given.

**Table 6:** Evaluation of the helium viscosity correlation for the primary data

Investigators (first author)/reference	AARD (%)	BIAS (%)	MAX (%)	AARD (%)	BIAS (%)	MAX (%)
	Present work			Arp et al. [9]		
Kochan-Eilers [25]	0.22	− 0.22	− 0.27	1.68	− 1.68	− 3.17
Sakoda [27]	0.25	0.07	− 0.52	0.21	− 0.04	− 0.61
Herrmann [21]	0.25	− 0.25	− 0.38	1.56	− 1.56	− 3.65
Seibt [28]	0.25	− 0.25	− 0.85	1.76	− 1.76	− 3.99
May [26]	0.05	0.05	0.05	0.04	− 0.04	− 0.05
Evers [18]	0.05	− 0.03	− 0.10	0.43	− 0.43	− 1.70
Wang [33]	0.80	− 0.05	1.97	7.62	− 6.19	− 25.18
Strehlow [25, 29]	0.05	0.03	0.12	0.06	− 0.05	− 0.10
Vogel [25, 32]	0.03	0.00	0.07	0.09	− 0.08	− 0.15
Kestin [24]	0.20	0.20	0.27	0.09	0.09	0.17
Karnus[22]	3.56	− 1.22	− 9.46	4.46	− 4.41	− 12.97
Kestin [23]	0.39	0.39	0.61	0.37	0.37	0.63
Gough [20]	0.52	0.52	1.59	0.46	0.44	1.45
Vermesse [31]	0.79	− 0.77	− 2.21	11.68	9.20	38.89
Goodwin [19]	6.52	0.63	13.68	12.27	− 12.07	− 22.18
Tsederberg [30]	3.22	3.22	6.89	3.94	− 3.68	-7.89
Kestin [56]	0.28	0.28	0.52	0.32	0.32	0.76
Webeler [57]	2.56	− 2.56	− 2.83	29.37	− 29.37	− 29.72
Kestin [58]	0.10	0.03	− 0.11	0.62	− 0.57	− 1.68
Steward [12]	3.93	1.42	− 12.92	3.78	1.16	− 15.04
Dawe [59]	1.21	− 1.11	− 2.36	0.75	− 0.69	− 1.11
Kalelkar [60]	0.59	− 0.21	− 2.10	0.47	− 0.10	− 1.49
Clarke [61]	0.61	0.61	1.22	0.71	0.25	− 2.31
Gracki [62]	0.29	− 0.04	1.00	2.61	− 2.61	− 6.27
Guevara [63]	1.04	− 0.87	− 3.86	1.05	0.65	1.41
DiPippo [64, 65]	0.17	0.17	0.52	0.09	0.07	0.52
DiPippo [66]	0.08	0.08	0.14	0.14	− 0.10	− 0.32
Kao [67]	0.40	− 0.30	− 1.02	3.29	− 3.29	− 7.48
Reynes [68]	0.68	0.41	1.34	1.54	− 1.54	− 2.68
Iwasaki [69]	0.12	− 0.05	− 0.27	0.49	− 0.47	− 1.14
Kestin [70]	0.17	0.05	0.69	0.54	− 0.45	− 1.53
Flynn [71]	0.24	− 0.15	− 0.53	2.23	− 2.23	− 5.27
Makavetskas [72]	1.85	1.05	4.55	1.19	− 0.09	− 2.11
Kestin [73]	0.11	− 0.09	− 0.22	0.44	− 0.44	− 1.29
Coremans [74]	3.93	3.93	6.26	0.99	− 0.64	− 2.52
Ross [75]	1.22	0.67	3.73	3.53	− 3.53	− 6.09
Becker [76]	3.75	3.75	5.17	1.57	− 1.52	− 2.56

**Table 7:** Evaluation of the helium viscosity correlation for the secondary data

Investigators (first author)/reference	AARD (%)	BIAS (%)	MAX (%)	AARD (%)	BIAS (%)	MAX (%)
	Present work			Arp et al. [9]		
Lukin [36]	1.01	0.98	1.67	0.98	0.50	− 2.06
Tanaka [39]	0.88	0.88	1.52	0.77	0.77	1.44
Vidal [40]	0.88	− 0.84	− 2.22	16.13	15.53	38.30
Kestin [35]	0.39	0.39	1.26	0.29	0.29	1.14
Kestin [78]	0.31	0.31	0.49	0.35	0.35	0.54
Maitland [37]	1.89	− 1.89	− 2.99	1.28	− 1.28	− 1.68
Rakshit [38]	0.47	0.07	− 0.81	0.43	− 0.01	− 0.87
Borisov [34]	0.96	− 0.96	− 0.96	1.05	− 1.05	− 1.05
Kestin [79]	0.39	0.39	0.68	0.44	0.44	0.81
Kestin [80]	0.10	0.10	0.15	0.11	− 0.09	− 0.25
Richardson [81]	0.43	− 0.35	− 0.72	0.99	− 0.95	− 1.54
Rudenko [82]	40.00	39.93	68.90	39.70	39.04	71.78
Kestin [83]	0.04	0.00	0.07	0.20	− 0.20	− 0.39
Golubev [84]	1.17	− 0.77	− 2.33	2.63	− 2.63	− 4.61
Webeler [85]	2.62	− 2.62	− 2.79	28.24	− 28.24	− 29.28
Kestin [86]	0.08	− 0.04	− 0.15	0.46	− 0.46	− 0.71
Stevenson [87]	0.84	− 0.25	− 0.9	0.89	− 0.35	− 1.00
Thornton [88]	1.07	− 1.07	− 1.58	1.15	− 1.15	− 1.66
Thornton [89]	0.59	− 0.59	− 0.59	0.68	− 0.68	− 0.68
Thornton [90]	0.59	− 0.59	− 0.59	0.68	− 0.68	− 0.68
Kestin [92]	0.10	0.08	0.23	1.04	− 1.04	− 1.85
Kestin [93]	0.20	− 0.03	0.43	0.73	− 0.72	− 2.38
Luker [94]	1.19	0.32	− 4.23	1.07	0.06	− 4.29
Rietveld [95]	1.64	1.64	2.83	0.89	− 0.62	− 2.52
Zinovieva [96]	6.09	− 6.09	− 8.62	12.84	− 12.84	− 29.07
Eisele [97]	16.41	− 6.60	31.62	18.36	− 11.84	− 41.02
Kestin [98]	0.08	0.08	0.31	0.24	− 0.24	− 0.48
Taylor [99]	8.43	8.43	14.63	8.60	− 5.00	− 21.59
Robinson [100]	18.46	15.86	75.70	35.75	7.47	92.16
Becker [101]	24.97	12.33	63.34	8.21	− 8.21	− 12.97
Kestin [102]	0.10	− 0.02	− 0.17	0.46	− 0.46	− 0.71
Rietveld [103]	2.81	1.27	8.12	1.66	− 0.08	5.18
Van Itterbeek [104]	31.04	30.96	408.64	7.63	7.63	24.43
Tjerkstra [105]	12.94	10.85	32.27	9.05	− 4.69	− 18.58
De Troyer [106]	19.74	− 19.74	− 24.75	25.28	− 25.28	− 34.60
Bowers [107]	11.19	8.32	22.35	17.95	13.89	− 36.85
Johnston [108]	30.04	29.13	59.33	29.92	28.59	59.19
Wobser [109]	0.27	− 0.27	− 0.39	0.37	− 0.37	− 0.51
Van Itterbeek [110]	1.83	1.83	3.99	1.29	− 0.77	− 3.38
Van Itterbeek [111]	29.02	− 27.48	− 95.90	30.12	− 29.88	− 95.99
Van Itterbeek [112]	23.57	− 16.54	− 96.76	24.19	− 19.78	− 96.93
Keesom [113]	24.54	− 24.54	− 40.81	30.36	− 30.36	− 52.50
Trautz [114]	19.10	− 19.10	− 55.38	20.74	− 20.74	− 55.41
Wilhelm [115]	162.01	162.01	284.37	144.03	144.03	229.83
Trautz [116]	1.25	− 1.25	− 1.71	1.35	− 1.35	− 1.79
Trautz [117]	1.39	− 1.39	− 2.28	1.49	− 1.49	− 2.38
Trautz [118]	1.25	− 0.84	− 2.44	1.30	− 0.95	− 2.54
Trautz [118]	2.68	− 2.68	− 4.63	2.53	− 2.53	− 4.09
Nasini [120]	0.78	0.78	1.00	0.68	0.68	0.88
Günther [121]	3.73	0.72	− 9.07	3.74	− 2.39	− 13.29
Ishida [122]	0.37	0.37	0.37	0.28	0.28	0.28
States [123]	0.50	− 0.50	− 0.50	0.58	− 0.58	− 0.58
Gille [124]	0.69	0.69	1.33	0.62	0.61	1.26
Vogel [125]	1.45	1.17	4.91	1.87	− 0.45	− 5.79
Kamerlingh Onnes [126]	2.75	2.75	4.59	1.33	− 0.18	− 3.07
Rankine [127]	0.03	0.03	0.03	0.05	− 0.05	− 0.05
Schmitt [128]	1.49	0.93	7.96	1.12	0.44	4.99

**Fig. 4:**
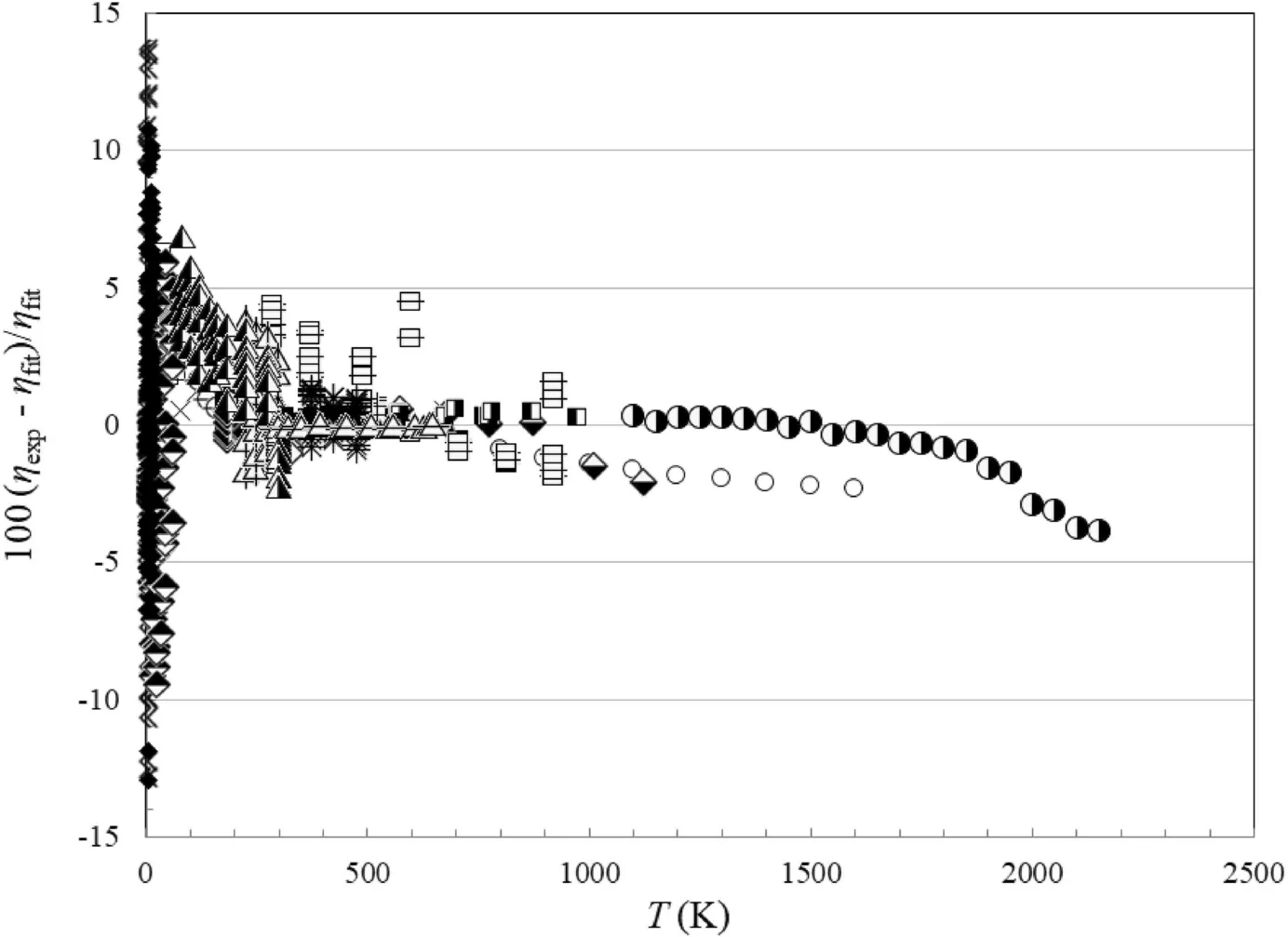
Percentage deviations of primary experimental data of helium from the values calculated by the present model as a function of temperature. Becker and Misenta [76] (+), Clarke and Smith [61] (×), Coremans et al. [74] (□) Dawe and Smith [59] (○), DiPippo et al. [66] (), DiPippo and Kestin [64, 65] (), Evers et al. [18] (), Goodwin [19] (), Gough et al. [20] (), Gracki et al. [62] (), Guevara et al. [63] (), Herrmann [21] (), Iwasaki and Kestin [69] (), Kalelkar and Kestin [60] (), Kao and Kobayashi [67] (), Karnus et al. [22] (), Kestin and Leidenfrost [73] (), Kestin and Whitelaw [70] (), Kestin et al. [58] (), Kestin et al. [56] (), Kestin et al. [23] (), Kestin and Wakeham [24] (), Kochan-Eilers et al. [25] (), Makavetskas and Popov [72] (), May et al. [26] (), Reynes and Thodos [68] (), Ross and Brown [75] (), Sakoda et al. [27] (), Seibt et al. [28] (−), Steward and Wallace [12] (), Strehlow [29] (), Tsederberg et al. [30] (), Vermesse and Vidal [31] (), Vogel [32] (), Wang et al. [33] (), and Webeler and Allen [57] ()

**Fig. 5:**
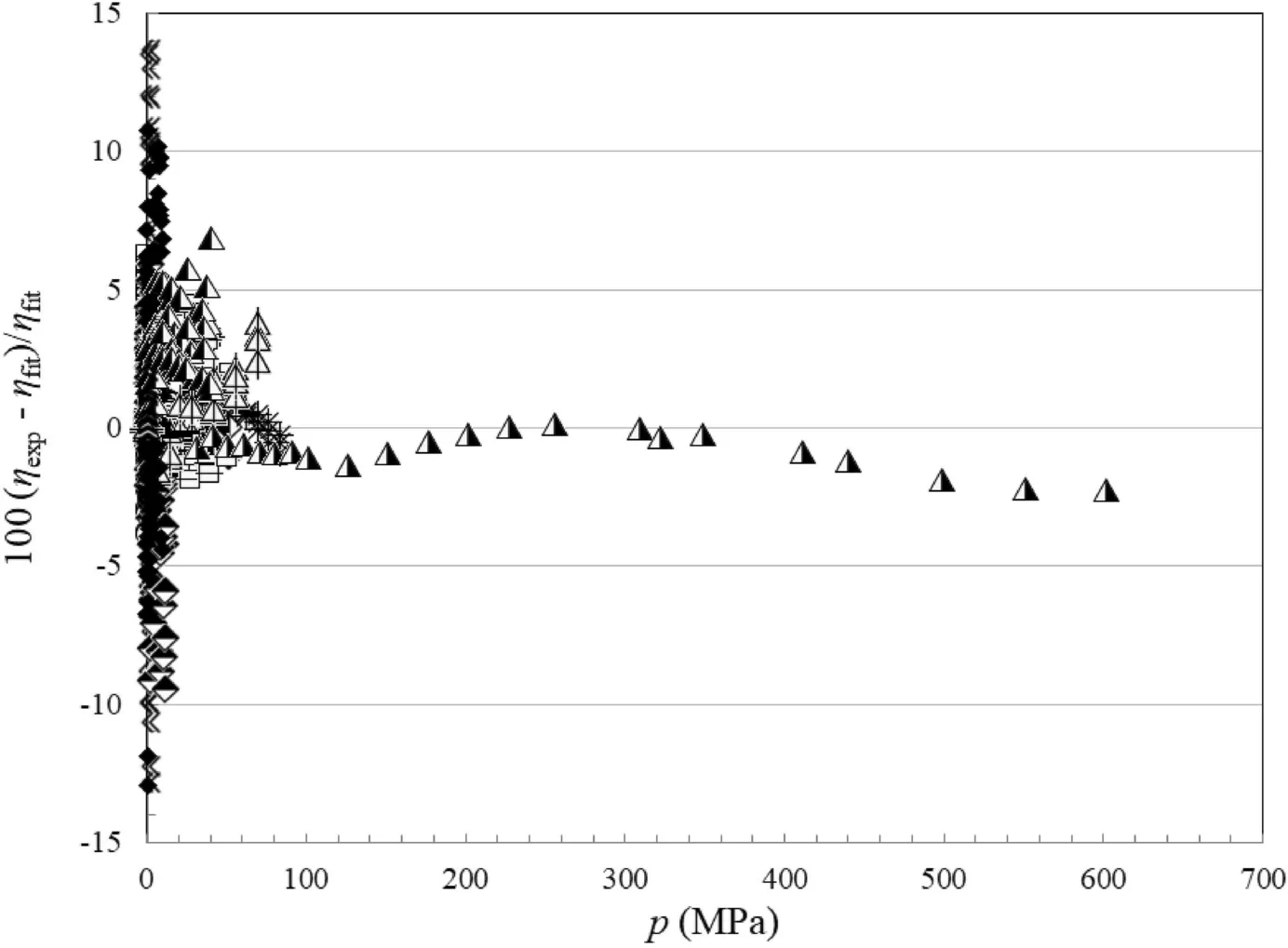
Percentage deviations of primary experimental data of helium from the values calculated by the present model as a function of pressure. Becker and Misenta [76] (+), Clarke and Smith [61] (×), Coremans et al. [74] (□) Dawe and Smith [59] (○), DiPippo et al. [66] (), DiPippo and Kestin [64, 65] (), Evers et al. [18] (), Goodwin [19] (), Gough et al. [20] (), Gracki et al. [62] (), Guevara et al. [63] (), Herrmann [21] (), Iwasaki and Kestin [69] (), Kalelkar and Kestin [60] (), Kao and Kobayashi [67] (), Karnus et al. [22] (), Kestin and Leidenfrost [73] (), Kestin and Whitelaw [70] (), Kestin et al. [58] (), Kestin et al. [56] (), Kestin et al. [23] (), Kestin and Wakeham [24] (), Kochan-Eilers et al. [25] (), Makavetskas and Popov [72] (), May et al. [26] (), Reynes and Thodos [68] (), Ross and Brown [75] (), Sakoda et al. [27] (), Seibt et al. [28] (−), Steward and Wallace [12] (), Strehlow [29] (), Tsederberg et al. [30] (), Vermesse and Vidal [31] (), Vogel [32] (), Wang et al. [33] (), and Webeler and Allen [57] ()

**Fig. 6:**
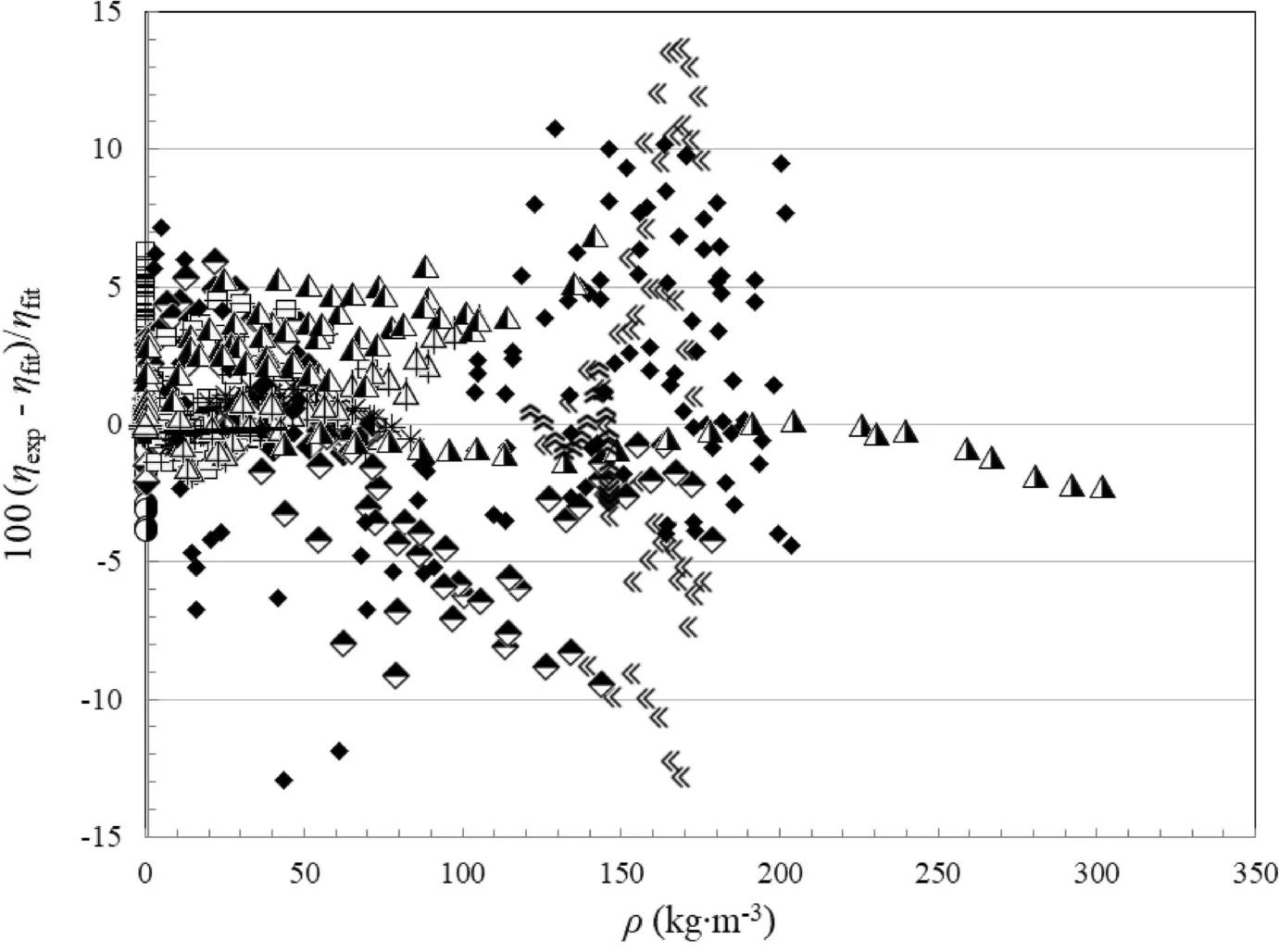
Percentage deviations of primary experimental data of helium from the values calculated by the present model as a function of density. Becker and Misenta [76] (+), Clarke and Smith [61] (×), Coremans et al. [74] (□), Dawe and Smith [59] (○), DiPippo et al. [66] (), DiPippo and Kestin [64, 65] (), Evers et al. [18] (), Goodwin [19] (), Gough et al. [20] (), Gracki et al. [62] (), Guevara et al. [63] (), Herrmann [21] (), Iwasaki and Kestin [69] (), Kalelkar and Kestin [60] (), Kao and Kobayashi [67] (), Karnus et al. [22] (), Kestin and Leidenfrost [73] (), Kestin and Whitelaw [70] (), Kestin et al. [58] (), Kestin et al. [56] (), Kestin et al. [23] (), Kestin and Wakeham [24] (), Kochan-Eilers et al. [25] (), Makavetskas and Popov [72] (), May et al. [26] (), Reynes and Thodos [68] (), Ross and Brown [75] (), Sakoda et al. [27] (), Seibt et al. [28] (−), Steward and Wallace [12] (), Strehlow [29] (), Tsederberg et al. [30] (), Vermesse and Vidal [31] (), Vogel [32] (), Wang et al. [33] (), and Webeler and Allen [57] ()

Figure 4 shows the percentage deviations of all primary viscosity data of helium from the values calculated by Eq. 8, as a function of temperature, while Figs. 5 and 6 show the same deviations but as a function of the pressure and the density. The quality of the fit is discussed in the sections below.

#### Low-Pressure Region (*p* ≤ 1 MPa)

**Fig. 7:**
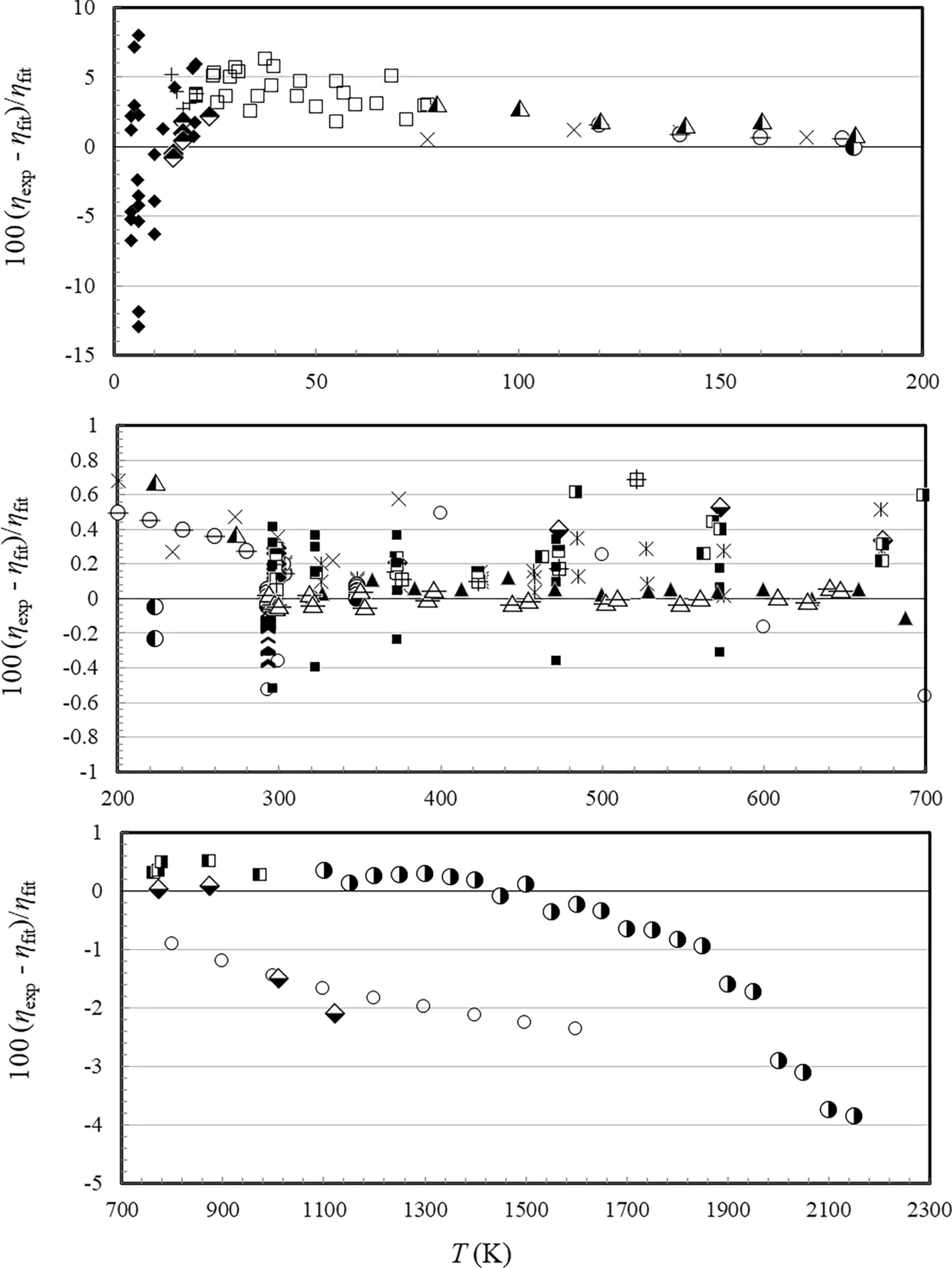
Percentage deviations of primary experimental data of helium from the values calculated by the present model as a function of temperature for the low-pressure region. Becker and Misenta [76] (+), Clarke and Smith [61] (×), Coremans et al. [74] (□), Dawe and Smith [59] (○), DiPippo et al. [66] (), DiPippo and Kestin [64, 65] (), Evers et al. [18] (), Goodwin [19] (), Gough et al. [20] (), Gracki et al. [62] (), Guevara et al. [63] (), Herrmann [21] (), Iwasaki and Kestin [69] (), Karnus et al. [22] (), Kalelkar and Kestin [60] (), Kao and Kobayashi [67] (), Kestin and Leidenfrost [73] (), Kestin and Whitelaw [70] (), Kestin et al. [58] (), Kestin et al. [56] (), Kestin et al. [23] (), Kestin and Wakeham [24] (), Kochan-Eilers et al. [25] (), Makavetskas and Popov [72] (), May et al. [26] (), Sakoda et al. [27] (), Seibt et al. [28] (−), Steward and Wallace [12] (), Strehlow [29] (), Tsederberg et al. [30] (), Vogel [32] ()

In the low-pressure region (defined here as pressures up to 1 MPa), numerous sources of experimental data are available, as outlined in Table 1. Figure 7 shows the deviations for all primary experimental data (excluding the liquid) at pressures up to 1 MPa as a function of temperature. The data coverage and quality vary across temperature, and Fig. 7 has been split into three temperature regions for ease of discussion. For temperatures below 14 K, there are only the data of Steward and Wallace [12] from 1971, which display a large amount of scatter. The correlation represents these data to about 10 %, which exceeds the estimated uncertainty of the data (2.5 % to 8 % with the highest uncertainty at the highest densities). From 14 K to almost 80 K there are additional data: Karnus et al. [22], Coremans et al. [74], and Becker and Misenta [76]. The data of Becker and Misenta [76] are consistent with Coremans et al. [74] and both sets display a positive bias of about 4 %, exceeding their estimated uncertainties. The data of Karnus and Rudenko [22] do not display a bias in this region and are represented to about 2 %. Near 80 K, there are additional data from Tsederberg et al. [30] and Clark and Smith [61]. Tsederberg et al. [30] show a positive bias of about 3 %, but the data of Clark and Smith [61] do not show any unusual bias. Additional experimental data in this region could help explain these discrepancies and allow refinement of the uncertainty estimates, as we will conservatively assign 4 % to the region between 50 K and 80 K. (All uncertainties are given at the 95 % confidence level; see Sect. 2.4.5 for discussion of uncertainties of the correlation). As the temperature increases above 80 K, the deviations decrease as the temperature increases to approximately 183 K. The data are sparse in this region, and agreements are generally within 2 %, but additional data would be beneficial. Between 200 K and 700 K, there are a large number of datasets; however, the most reliable data in this regime span the temperature range from 294 K to 688 K, specifically the measurements of Vogel [32] and Strehlow [29], both of which were recently re-analyzed with an updated calibration [25]. The reported experimental uncertainty for these sets is 0.2 % between 294 K and 400 K, and 0.4 % between 400 K and 688 K. As shown in Fig. 7, the correlation agrees with these datasets well within their estimated experimental uncertainties; comparisons in Table 6 indicate an agreement of 0.11 % (at *k* = 2) with Strehlow [25, 29] and 0.07 % with Vogel [25, 32]. Note that these measurements were taken at extremely low densities (maximum 0.03 mol·L^-1^). The measurements of May et al. [26] also are of very low uncertainty; these points are represented to within 0.05 %, but there are only 2 points at 298.15 K. Under these conditions, the viscosity is dominated by the dilute gas contribution, which in our model is based on theoretical calculations that are significantly more accurate than physical experiments. This will be discussed in more detail later. The set of Evers et al. [18] contains data from 293 K to 348 K at pressures from 0.12 MPa to 9.5 MPa, and the entire set is represented to within the experimental uncertainty of 0.14 % to 0.4 %. Finally, at the highest temperatures, above 700 K, the data are sparser and show large deviations, but again in this region, the viscosity is dominated by the zero-density contribution.

#### Mid-Pressure Region (1 MPa < *p* < 100 MPa)

**Fig. 8:**
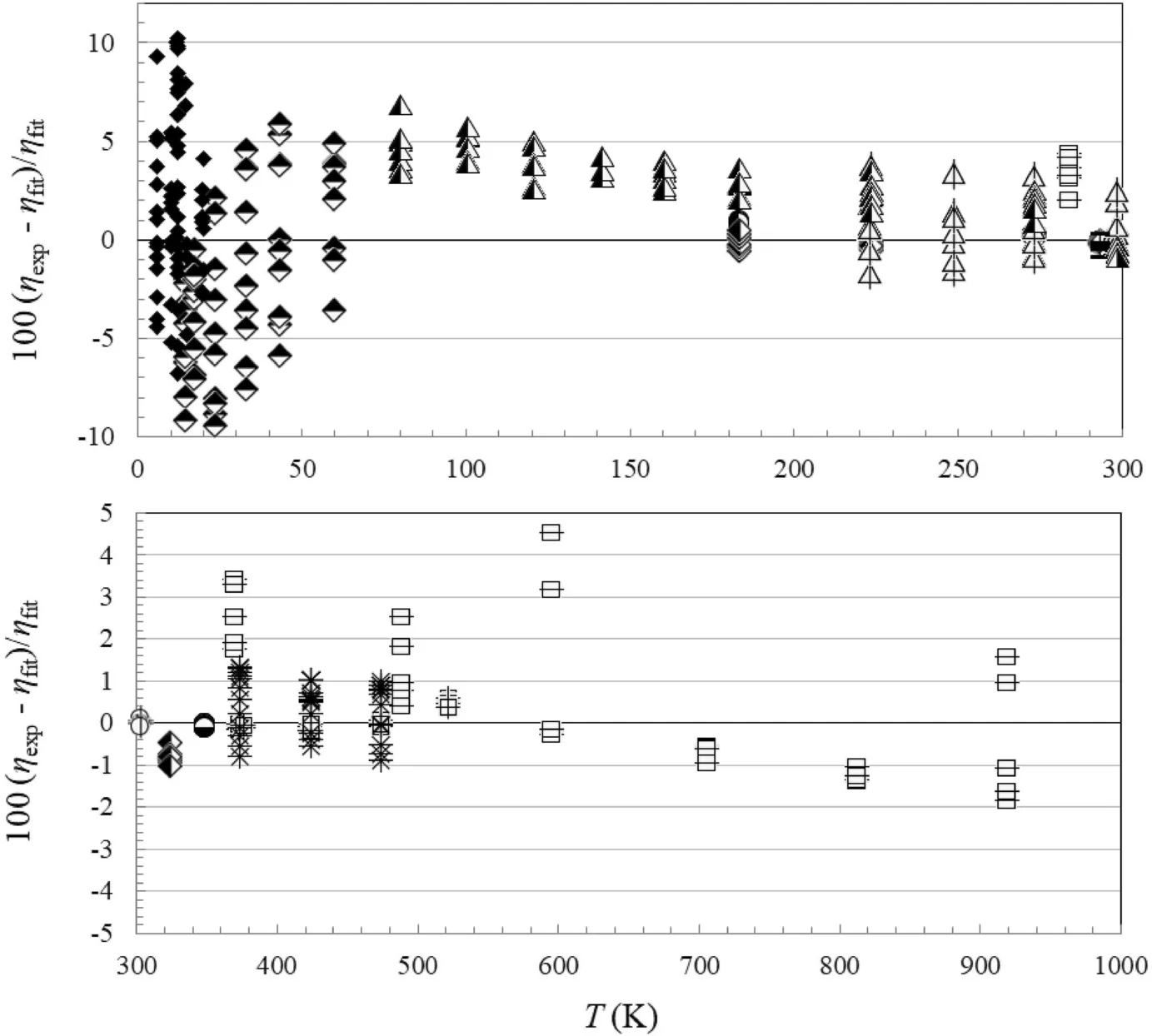
Percentage deviations of primary experimental data of helium from the values calculated by the present model as a function of temperature for the mid-pressure region. DiPippo and Kestin [64, 65] (), Evers et al. [18] (), Gracki et al. [62] (), Guevara et al. [63] (), Herrmann [21] (), Iwasaki and Kestin [69] (), Kao and Kobayashi [67] (), Karnus et al. [22] (), Kestin and Leidenfrost [73] (), Kestin and Whitelaw [70] (), Kestin et al. [58] (), Kochan-Eilers et al. [25] (), Makavetskas and Popov [72] (), Reynes and Thodos [68] (), Ross and Brown [75] (), Seibt et al. [28] (−), Steward and Wallace [12] (), Tsederberg et al. [30] (), Vermesse and Vidal [31]

Figure 8 shows the deviations as a function of temperature in the mid-pressure region (1 MPa to 100 MPa). It is split into 2 panels, one for temperatures below 300 K and the other for higher temperatures. For the very lowest temperatures, below 14 K, there are only the data of Steward and Wallace [12] that have deviations up to 10 %. At slightly higher temperatures, in addition to a few points from Steward and Wallace [12], there also are data from Karnus et al. [22], and the deviations in this region are slightly lower, averaging about 8 %. Starting at 80 K until 160 K, the data of Tsederberg et al. [30] show a systematic positive bias of 3 % to 4 % and are the only data available in this temperature range. At about 180 K. the Tsederberg et al. [30] data can be compared with Kao and Kobashi [67] and Gracki et al. [62]. The data of Kao and Kobayashi [67] were taken with a capillary viscometer, and for the 183 K isotherm, the pressures span 1 MPa to 20 MPa; the maximum deviation of this isotherm is 0.5 % and a systematic bias is not apparent. The entire dataset that spans 183 K to 273 K at pressures up to 51 MPa is represented to within 0.7 % (at *k* = 2). Similarly, the isotherm of Gracki et al. [62] at 183 K extends to pressures of 17 MPa with a maximum deviation of 1 % and does not show any unusual systematic deviations. We do not have an explanation for the bias in the data of Tsederberg et al. [30]. In the temperature range starting at 223 K up to 373 K covering pressures 2.2 MPa to 17.8 MPa, Flynn et al. [71] provide an additional high-quality data source for comparisons. The Flynn et al. [71] data are all represented to within 0.5 %. Some other sources of high-quality data in the mid-pressure range are Kochan-Eilers et al. [25], Herrmann [21], Seibt et al. [28], and Kestin et al. [58] but each only provides data at a single temperature (293 K, 293 K, 298 K, and 298 K, respectively) with a maximum pressure of 21 MPa, 25.3 MPa, 30 MPa, and 10 MPa, respectively. These sets are all fitted very well by the model, to within their estimated uncertainties. Kestin and Whitelaw [70] made measurements with an estimated uncertainty of 0.5 % over the temperature range 296 K to 521 K at pressures from 0.14 MPa to 12 MPa. The model represents these data to within the experimental uncertainty. At pressures above 50 MPa, the data of Reynes and Thodos [68] cover temperatures from 373 K to 473 K and are represented to within 1 %. At temperatures above 473 K and pressures above 1 MPa, the only dataset available is Makavetskas and Popov [72] that has an uncertainty of 3.5 % and covers the region from 4 MPa to 51 MPa at temperatures up to 918 K. At a *k* = 2 level of uncertainty, this dataset is represented to 4 %. Additional low-uncertainty data in this region would be helpful for further validation. The data of Verness and Vidal [31] extend to 601 MPa but are only available at a single temperature of 298 K; at pressures up to 100 MPa, they are represented to within about 0.5 %.

#### High-Pressure Region (*p* > 100 MPa)

**Fig. 9:**
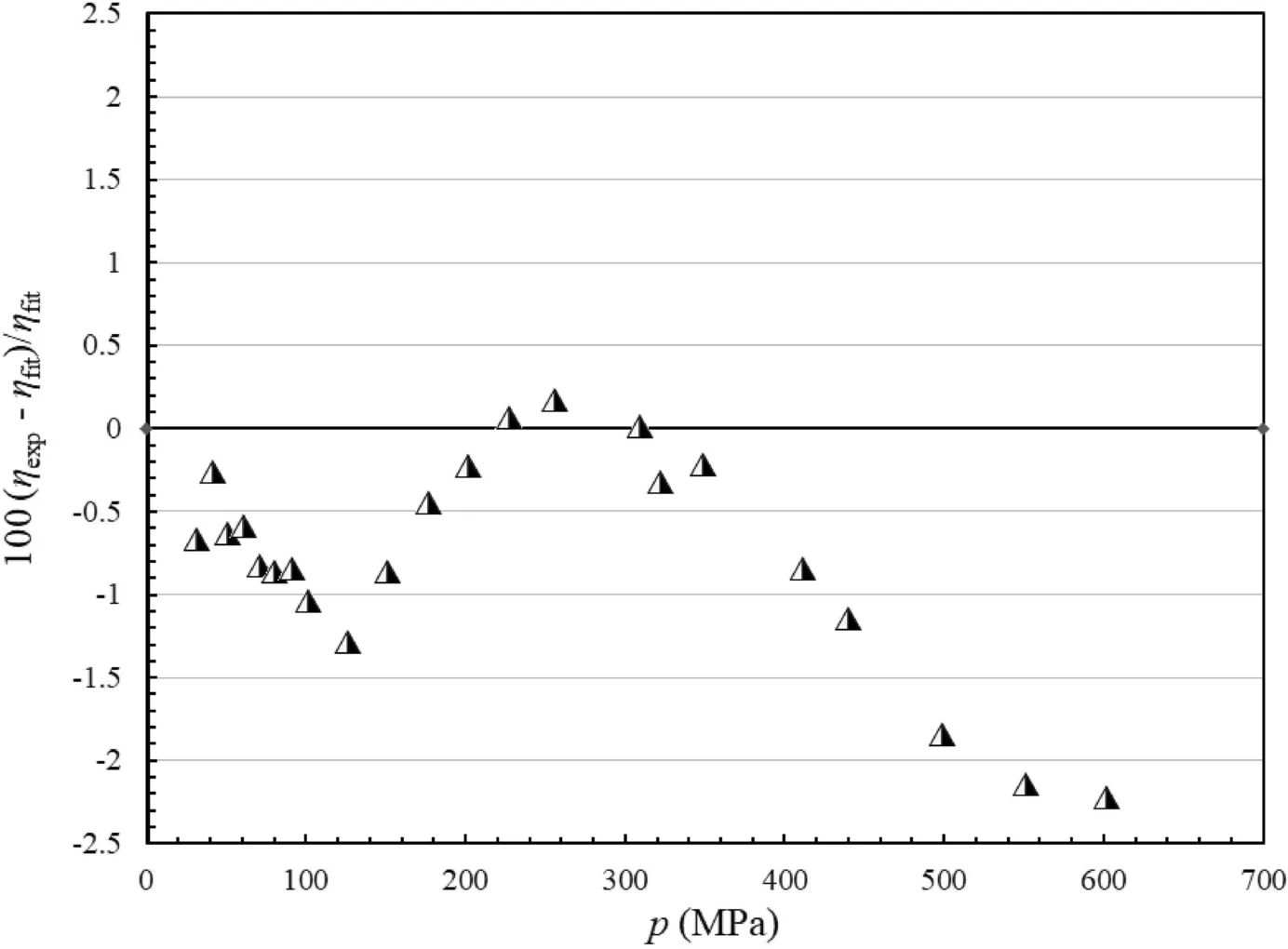
Percentage deviations of primary experimental data of helium from the values calculated by the present model as a function of pressure for the high-pressure region. Vermesse and Vidal [31] ()

There is only one dataset that extends to pressures above 100 MPa that of Vermesse and Vidal [31]. Unfortunately, these data are limited to 298 K. Based on comparisons with very limited data, the region above 100 MPa, the estimated relative expanded (*k* = 2) uncertainty of the correlation is 1.5 %, although the deviations show a systematic, increasingly negative trend at pressures above 300 MPa. Additional high-pressure data are needed to fully validate the model in this region (Fig. 9).

#### Liquid Phase

**Fig. 10:**
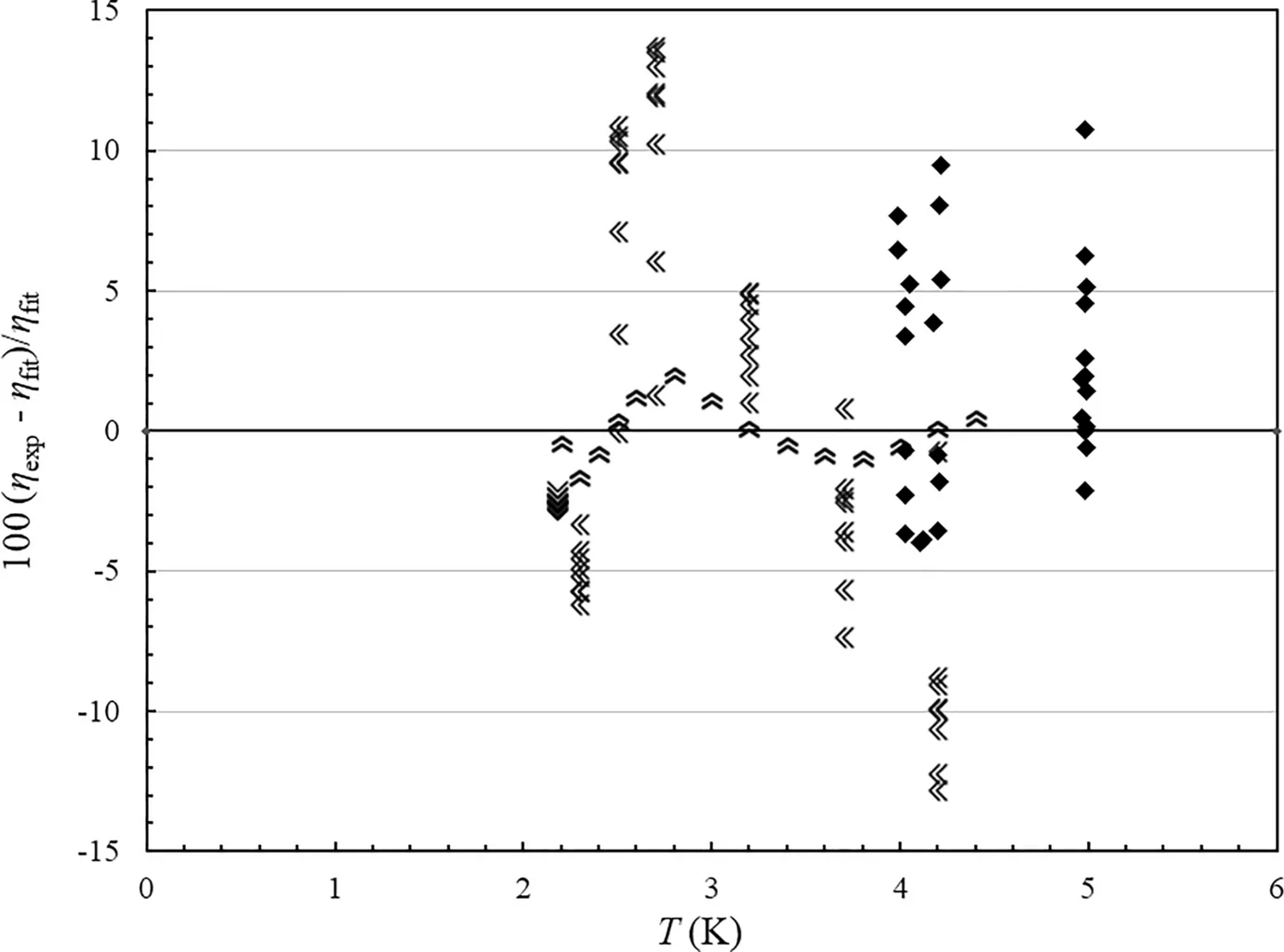
Percentage deviations of primary experimental data of helium from the values calculated by the present model as a function of temperature for the liquid region. Goodwin [19] (), Steward and Wallace [12] (), Wang et al. [33] (), and Webeler and Allen [57] ()

**Fig. 11:**
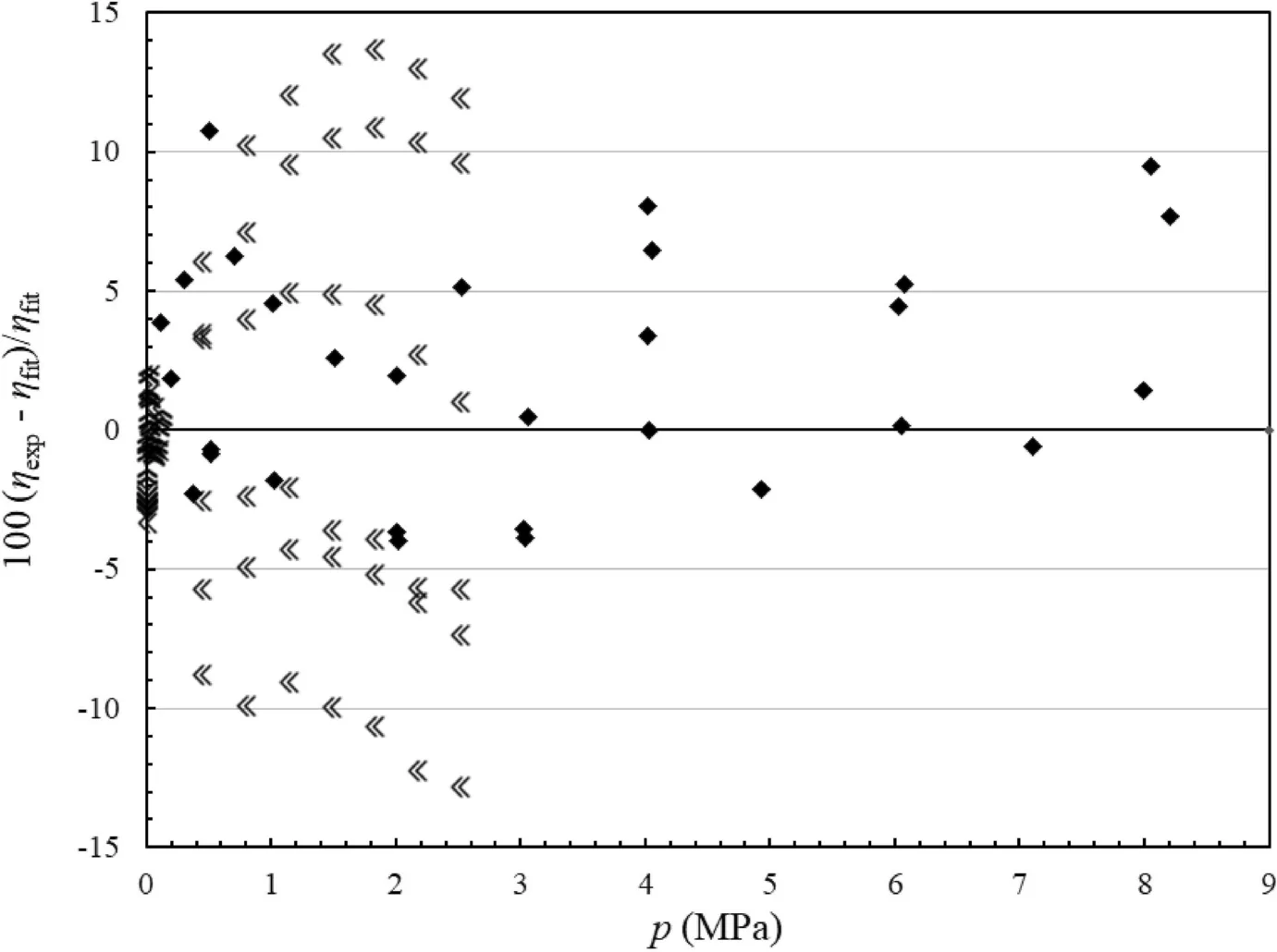
Percentage deviations of primary experimental data of helium from the values calculated by the present model as a function of pressure for the liquid region. Goodwin [19] (), Steward and Wallace [12] (), Wang et al. [33] (), and Webeler and Allen [57] ()

There are four primary data sources for the liquid phase [12, 19, 33, 57] and two of them [33, 57] are limited to the saturation boundary. The data of Webeler and Allen [57] along the saturation boundary are all at basically the same temperature very near the lambda point, have an estimated uncertainty of 8 %, and are presented only graphically in the original manuscript. The data of Wang [33] cover a wider range of temperatures along the saturation boundary, 2.2 K to 4.4 K, and have an estimated uncertainty of 4 %. The deviations of the correlation from the data are all within this level of uncertainty. For liquid pressures above saturation, the data from Steward and Wallace [12] contain 30 liquid points over the temperature range between 4 K and 5 K at pressures up to 8 MPa. All deviations are within 10 %, slightly exceeding being within their estimated uncertainty of 2 % to 8 %. The liquid-phase measurements of Goodwin [19] cover temperatures from 2.3 K to 4.2 K and pressures up to 2.5 MPa. They have an estimated uncertainty of 5 % to 6 %, and the model does not represent the data to within that level of uncertainty. Additional measurements in the liquid phase would be helpful to improve the model in this region (Figs. 10, 11).

#### Uncertainties

We generally estimate the uncertainty of a correlation by comparison with the most accurate experimental data available, and we will do so here. However, the dilute gas viscosity of helium from ab initio calculations has a much lower uncertainty than any currently available experimental measurements, and this information can be leveraged to provide uncertainty estimates for the low-density gas that are lower than the uncertainties that can be obtained through experiment. For slightly higher densities, in the moderately dense gas region, Berg and Moldover [141] did an extensive analysis using data from [18, 62, 71, 73, 86] and reported a value for the second viscosity virial coefficient *B*_*η*_ (see Eq. 5) of − 1.1 × 10^−4^ ± 1.3 × 10^−4^ m^3^·kg^−1^, where they report standard uncertainties (*k* = 1). Although the relative uncertainty in *B*_*η*_ is very large (more than 100 %), its absolute contribution to the total viscosity uncertainty at 298.15 K and 0.1 MPa is extremely small.

For these low densities where contributions of Δ*η*_high_ (Eq. 7) are negligible, the combined standard uncertainty of the total viscosity can be expressed using the root-sum-square method as

12 $$ u_{\eta } (T,\rho ) = \sqrt {u_{{\eta_{0} }}^{2} + u_{{{\mathrm{linear}}}}^{2} } , $$

where $u_{{\eta_{0} }}$ is the standard uncertainty in the zero-density term and $u_{{{\mathrm{linear}}}}$ is the propagated uncertainty in the linear density correction term. The uncertainty of the zero-density term is provided in Cencek et al. [14], and the uncertainty in the linear-in-density term in Eq. 8 can be expressed as

13 $$ u_{{{\mathrm{linear}}}} = \eta_{0} \rho u_{{B_{\eta } }} , $$

where $u_{{B_{\eta } }}$ is the standard uncertainty in *B*_*η*_. Using a density of helium at 298.15 K and 0.1 MPa as 0.161386 kg·m^−3^ (from the EOS of Ortiz-Vega et al. [53]), and the Berg and Moldover recommended values for *η*_0_ and *B*_*η*_, one calculates a viscosity at 298.15 K and 0.1 MPa of 19.8250 ± 0.0005 μPa·s, or a relative standard uncertainty of 0.0023 % which is much lower than uncertainties in the experimental values for the first density correction.

We can apply similar logic to obtain an estimate of the uncertainty for the viscosity correlation developed here, as long as *B*_*η*_ has not entered the very low-temperature region where it rapidly goes negative. In that region, we have no reliable method to estimate the uncertainty of the linear-in-density term, so we will have to rely solely on comparisons with experimental data. At higher temperatures, we can estimate that the uncertainty of the linear-in-density term is that given by Berg and Moldover at 298.15 K, but will scale linearly with density, as shown in Eq. 13. However, we must first include an additional term to account for terms of higher order than linear. Our model contains a higher-order empirical term, Eq. 7, that is a 13-term polynomial function of reduced temperature and density, with the lowest density dependence being $\rho_{{\mathrm{r}}}^{1.25}$. To determine the total combined standard uncertainty, the root-sum-square method is expanded to incorporate the uncertainty of this higher-order term, *u*_high_

14 $$ u_{\eta } (T,\rho ) = \sqrt {u_{{\eta_{0} }}^{2} + u_{{{\mathrm{linear}}}}^{2} + u_{{{\mathrm{high}}}}^{2} } . $$

Because the empirical coefficients of the residual function are highly correlated, propagating uncertainty through the individual terms is impractical. Instead, we will adopt a conservative estimate, designating a 100 % relative uncertainty to the total magnitude of the residual contribution. (Recall the relative uncertainty on the second viscosity virial coefficient was ~ 100 %). Therefore, the standard uncertainty of the higher-order term is simply equal to its absolute computed value,

15 $$ u_{{{\mathrm{high}}}} = \left| {\Delta \eta_{{{\mathrm{high}}}} } \right|. $$

Applying this to the point at 298.15 K and 0.1 MPa, we get a viscosity of 19.8284 ± 0.0043 μPa·s, or a relative standard uncertainty of 0.022 %, almost an order of magnitude larger than the reference value of Berg and Moldover, but still considerably lower than a typical experimental uncertainty of 0.1 % to 0.2 %. The values of viscosity of our correlation and the Berg and Moldover point agree (at *k* = 1) to within their uncertainties. Vogel also provided a recommended value for the viscosity in the limit of zero density and the initial density dependence. He recommended the zero-density value of Cencek et al. [14] and an initial density dependence (∂*η*/∂*ρ*)_*T*_ of − 5.878  μPa·s·m^3^·kg^−1^ resulting in a value of viscosity at 298.15 K and 0.1 MPa of 19.82438 μPa·s that lies within the *k* = 2 band of our correlation.

As fluid density increases, the empirical higher-order residual term becomes a significant contributor to the total viscosity. Since it is purely empirical, a conservative estimate of 100 % relative uncertainty was applied to its magnitude. Consequently, the combined standard uncertainty grows rapidly in the dense gas and supercritical regions, eventually exceeding typical experimental uncertainties. To address this, we employ a hybrid method for establishing uncertainties: the theoretical uncertainty calculation discussed above is used where it yields an uncertainty lower than the best experimental data; once that threshold is exceeded, we switch to bounding the uncertainty using comparisons with experimental data.

**Fig. 12:**
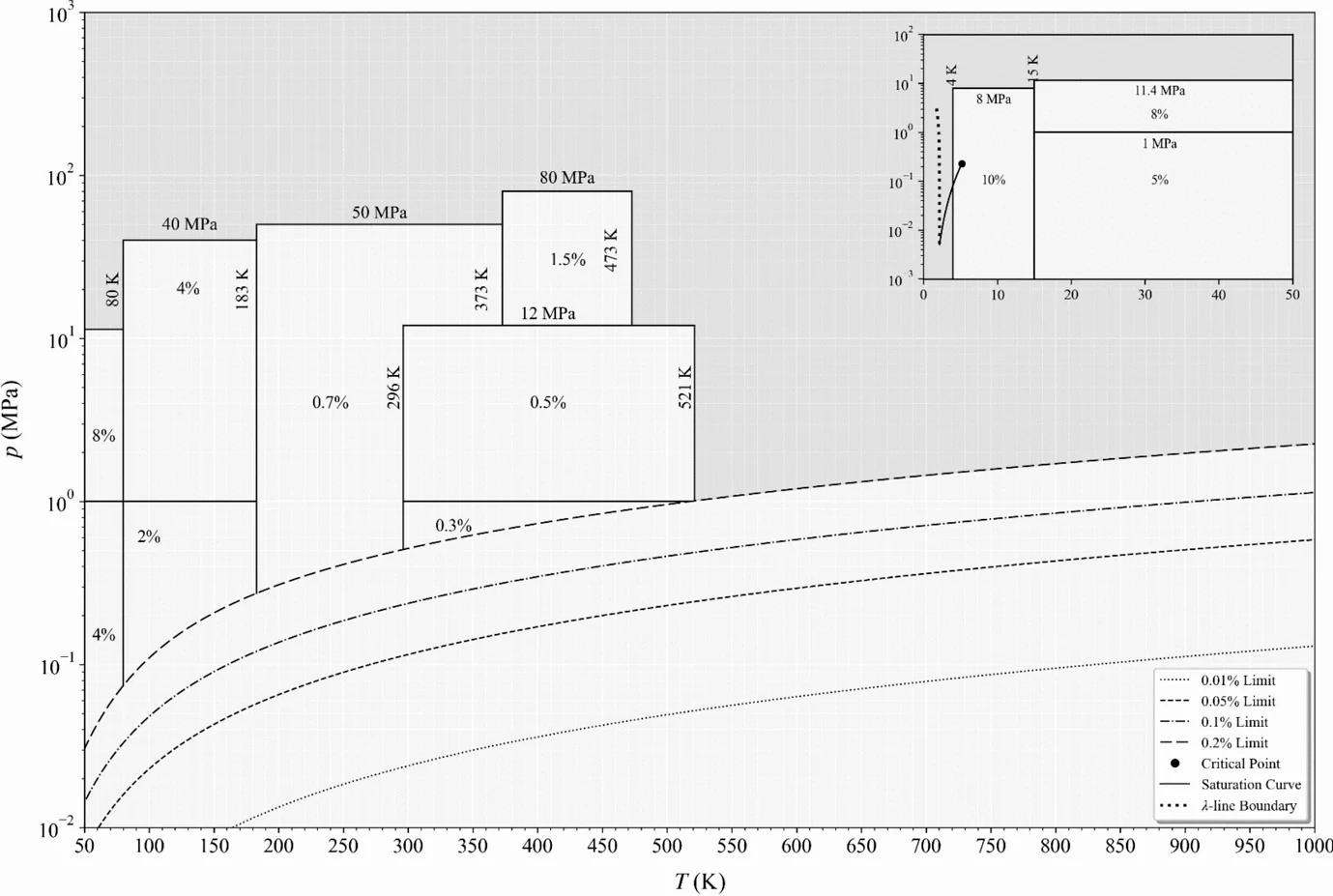
Estimated uncertainties (*k* = 2) for the correlation in different temperature–pressure regions

Figure 12 summarizes these expanded uncertainties (*k* = 2), illustrating the theoretically calculated regions, the experimentally bounded regions, and shaded areas where adequate experimental data for comparison are lacking. We note that even in some regions where there are some experimental data, such as for temperatures below 100 K, additional data are needed, since the data are sparse and two sources [12, 22] are presently difficult to obtain. For visual clarity, the 4 % estimated uncertainty for the saturated liquid viscosity is omitted from the inset in Fig. 12. The supplemental information includes files containing the *T,p* coordinate locations for the calculated 0.01 %, 0.05 %, 0.10 %, and 0.2 % uncertainty boundaries (at *k* = 2). Additionally, a Python script (helium_viscosity_uncertainty.py), designed for use with the NIST REFPROP program, is provided. This script calculates the viscosity and associated relative expanded uncertainty strictly for points within the theoretical (i.e., the low-pressure region in which theoretically calculated viscosities form the basis of the correlation) regime (uncertainties up to 0.2 % at *k* = 2). For states outside this regime, the region-specific experimental limits detailed in Fig. 12 should be applied.

**Fig. 13:**
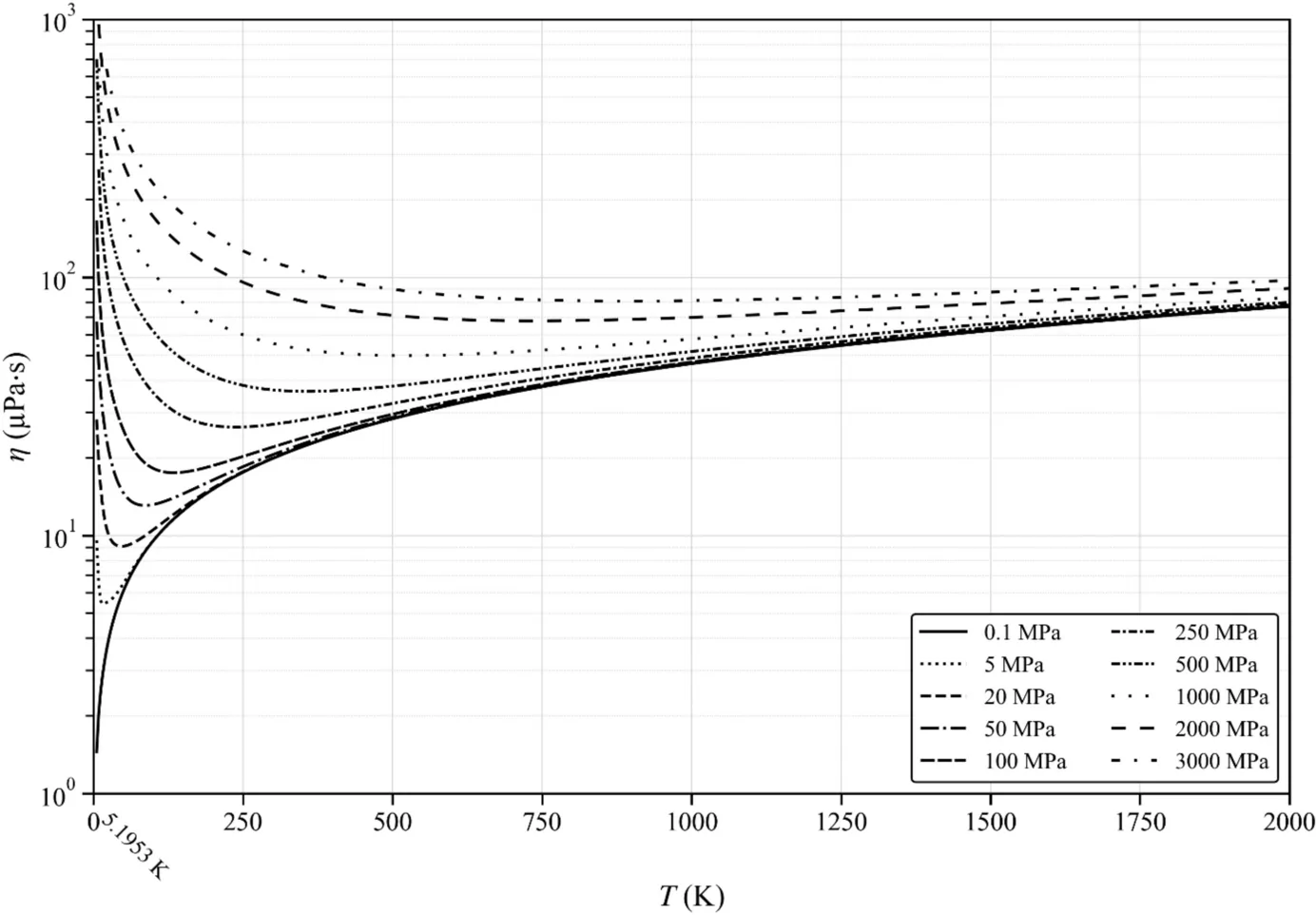
Viscosity of helium as a function of the temperature along isobars, for the supercritical fluid

**Fig. 14:**
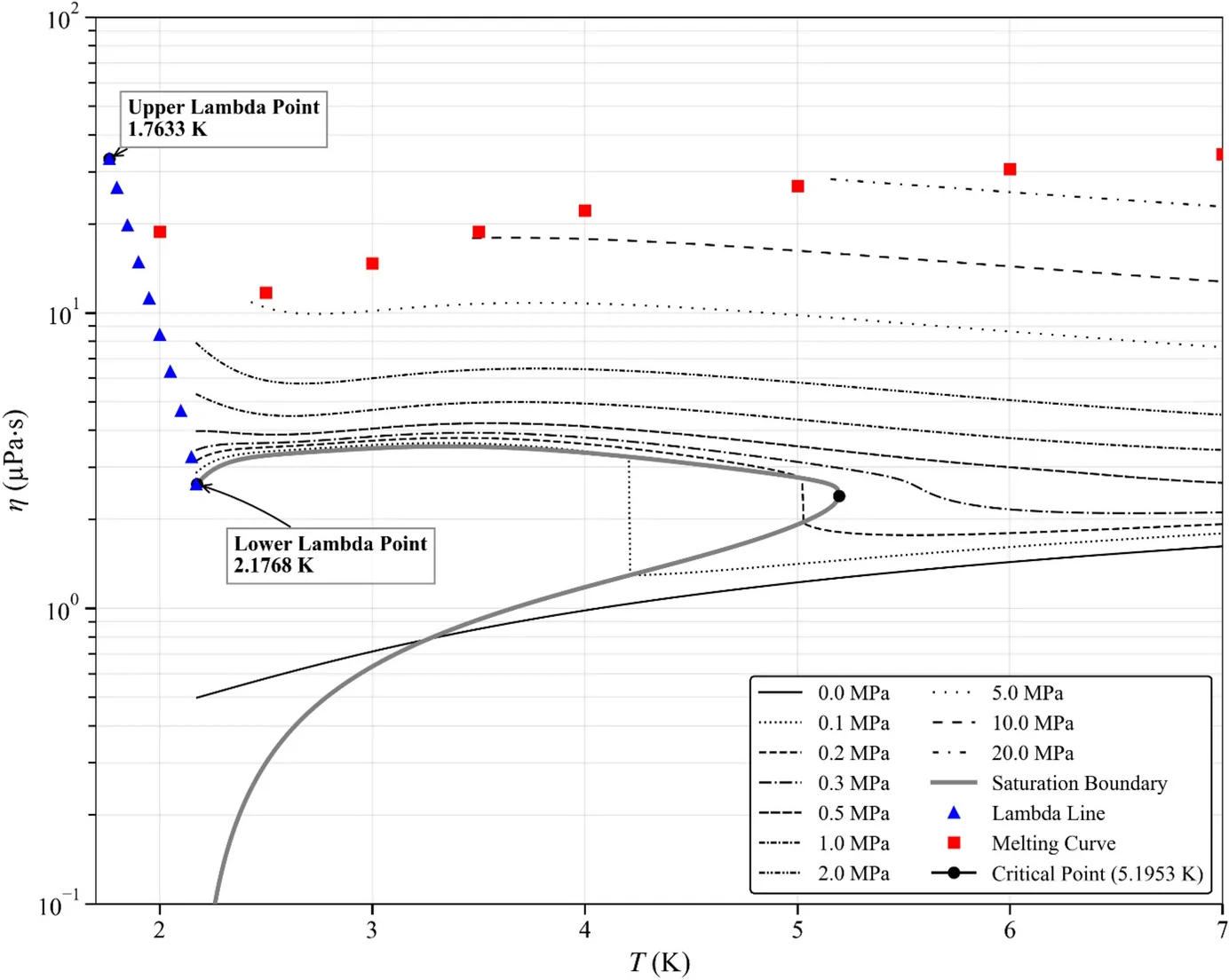
Viscosity of helium as a function of the temperature along isobars at low temperatures

Figure 13 shows a plot of the viscosity of helium as a function of the temperature for different pressures in the supercritical region, extrapolating to regions beyond the range (1500 K and 2000 MPa) of the EOS of Ortiz-Vega et al. [53] The behavior is physically reasonable without mathematical poles or negative viscosities. It is also interesting to look at the low-temperature behavior of the correlation in more detail. Figure 14 shows the viscosity of helium as a function of the temperature along isobars at low temperatures**.** Values for the viscosity along the lambda line and the melting curve are shown. These values were generated by using the temperature and density of the lambda line and the melting line as given by McCarty [143] and then using the correlation to obtain the viscosity.

The rise of the viscosity near the lambda line seems to mimic the behavior of more typical fluids upon approach to the melting line, and this rise with lower temperatures is seen for the supercritical fluid in Fig. 13. To our knowledge, the behavior shown above the lambda line has not been documented experimentally. Certainly, when the lambda line is crossed (temperature lowered), the flow behavior of liquid helium changes dramatically, and the measured viscosity can depend on the experimental method used. Further discussion of superfluid viscosity and approach to superfluidity is beyond the scope of the present work.

## Recommended Values and Computer Program Verification

### Recommended Values

In Table 8, viscosity values are given along the saturation boundary, calculated from the present proposed correlation between 2.5 K and 5.0 K. Liquid and vapor values for the tabulated densities and viscosities in Table 8 are indicated by the subscripts “liq” and “vap,” respectively. In Table 9, viscosity values are given for temperatures between 25 K and 2000 K and at selected pressures. Saturation density values for the selected temperatures in Table 8, as well as the density values for the selected temperature and pressure in Table 9 are obtained from the equation of state of Ortiz-Vega et al. [53]. The viscosity values in the tables are determined from the selected temperatures and calculated densities according to Eq. 8.

**Table 8:** Viscosity values of helium along the saturation boundary, calculated by the present scheme

*Τ* (Κ)	*ρ*_liq_ (kg·m^−3^)	*ρ*_vap_ (kg·m^−3^)	*η*_liq_ (μPa·s)	*η*_vap_ (μPa·s)
2.5	144.88	2.1395	3.2685	0.29902
3.0	141.20	4.4535	3.4718	0.63451
3.5	135.90	8.0656	3.5188	0.91478
4.0	128.74	13.548	3.3596	1.1778
4.5	118.49	22.255	3.0868	1.4703
5.0	99.841	39.707	2.7718	1.9184

**Table 9:** Viscosity values of helium at selected temperatures and pressures, calculated by the present scheme

*p* (MPa)	*T* (K)	*ρ* (kg·m^−3^)	*η* (μPa·s)
0.1	25	1.9246	3.9347
	50	0.96081	6.1061
	100	0.48073	9.563
	150	0.32062	12.491
	200	0.24053	15.134
	300	0.16039	19.912
	500	0.096255	28.331
	750	0.064176	37.709
	1000	0.048135	46.354
	2000	0.024069	77.248
10	25	128.97	6.9943
	50	76.232	7.3958
	100	42.116	9.7741
	150	29.255	12.515
	200	22.445	15.136
	300	15.328	19.939
	500	9.3815	28.393
	750	6.316	37.776
	1000	4.7595	46.413
	2000	2.3955	77.269
50	25	242.23	21.477
	50	198.42	14.624
	100	141.09	13.190
	150	109.32	14.527
	200	89.325	16.427
	300	65.477	20.589
	500	42.697	28.708
	750	29.729	38.012
	1000	22.786	46.621
	2000	11.754	77.386
100	25	290.69	40.996
	50	256.97	25.601
	100	205.30	18.204
	150	169.66	17.612
	200	144.65	18.674
	300	111.96	22.045
	500	77.191	29.530
	750	55.552	38.540
	1000	43.343	47.016
	2000	22.982	77.578
200	25	341.31	77.448
	50	312.85	46.959
	100	272.49	29.889
	150	240.69	24.964
	200	214.90	23.801
	300	176.77	25.237
	500	130.80	31.496
	750	98.763	39.926
	1000	79.248	48.090
	2000	44.036	78.101
500	25	410.81	166.29
	50	384.68	99.713
	100	354.06	61.806
	150	331.56	48.014
	200	312.54	41.466
	300	280.39	36.679
	500	231.27	37.936
	750	189.04	44.365
	1000	159.77	51.676
	2000	98.248	80.172
1000	25	464.06	275.47
	50	439.69	167.69
	100	412.66	104.08
	150	394.17	79.844
	200	379.23	67.301
	300	354.65	55.523
	500	315.72	49.839
	750	277.06	52.301
	1000	245.93	57.733
	2000	167.86	83.805

### Computer Program Verification

For checking computer implementations of the correlation, the following points may be used for the given *T*, *ρ* conditions: *T* = 298.15 K, *ρ* = 0 kg·m^−3^, *η* = 19.8253 μPa·s, *T* = 298.15 K, *ρ* = 0.16139 kg m^−3^ (*p* = 0.1 MPa), *η* = 19.8284 μPa·s, *T* = 298.15 K, *ρ* = 1.607 kg·m^−3^ (*p* = 1 MPa), *η* = 19.8470 μPa·s, and *T* = 298.15 K, *ρ* = 112.43 kg·m^−3^ (*p* = 100 MPa), *η* = 21.9777 μPa·s. A sample Python program to assist in calculating these points, helium_viscosity_check_points.py, is included in the Supplemental Information.

## Conclusions

We present a new, wide-ranging correlation for the viscosity of the normal fluid (helium I) based on critically evaluated experimental data and ab initio calculations. This correlation is designed to be used over the range of applicability of the equation of state of Ortiz-Vega et al. [53] and extends from the low-pressure endpoint of the *λ* curve on the vapor–liquid saturation boundary (2.1768 K) to 1500 K for pressures up to 2000 MPa. We leverage highly accurate theoretical calculations of Cencek et al. [14] for the viscosity of the gas in the zero-density limit in order to obtain an estimated relative expanded uncertainty (*k* = 2) of 0.044 % at 298.15 K and 0.1 MPa. To establish uncertainties across the entire fluid surface, we employed a hybrid approach: theoretical bounds were applied where they superseded experimental accuracy, while comparisons with primary experimental data were used to bound the uncertainty in denser regions where the empirical higher-order residual terms dominate. However, in regions where experimental data are sparse or of limited accuracy, the uncertainties remain large. The regions most in need of new experimental data are the low-temperature gas phase (4 K to 180 K, pressures up to 50 MPa) and the liquid phase (2.2 K to 5 K, pressures up to 50 MPa), where existing data are sparse, difficult to obtain, and have uncertainties of 2 % to 8 %. New low-uncertainty measurements (1 % or better) in these regions would most significantly reduce the correlation uncertainties, which are currently as large as 10 % in the liquid phase and 4 % to 8 % in the low-temperature gas phase.

## Supplementary Information

Below is the link to the electronic supplementary material.

Supplementary file1 (ZIP 52 kb)—The supporting information contains a text file, HELIUM.fld, for use with the NIST REFPROP v10 computer program. A sample python implementation of the correlation that provides the viscosity and estimated uncertainty for the region where the uncertainty is up to 0.2 %, helium_viscosity_uncertainty.py, is included, and the T,p coordinates for the 0.01, 0.05, 0.1 and 0.2 % uncertainty boundaries as plain text files. Running the python program helium_viscosity_uncertainty.py requires that the NIST REFPROP program be installed. A simpler program that reproduces the check points and does not require additional programs, Helium_viscosity_check.points.py, is also included to assist in programming the correlation.

## Data Availability

No datasets were generated or analyzed during the current study.
